# Amycolachromones A–F, Isolated from a Streptomycin-Resistant Strain of the Deep-Sea Marine Actinomycete *Amycolatopsis* sp. WP1

**DOI:** 10.3390/md20030162

**Published:** 2022-02-24

**Authors:** Jianwei Chen, Jun Chen, Siqi Wang, Xiaoze Bao, Songwei Li, Bin Wei, Huawei Zhang, Hong Wang

**Affiliations:** 1 College of Pharmaceutical Science & Collaborative Innovation Center of Yangtze River Delta Region Green Pharmaceuticals, Zhejiang University of Technology, Hangzhou 310014, China; cjw983617@zjut.edu.cn (J.C.); 2112007272@zjut.edu.cn (S.W.); baoxiaoze@zjut.edu.cn (X.B.); songweili93@zjut.edu.cn (S.L.); binwei@zjut.edu.cn (B.W.); hwzhang@zjut.edu.cn (H.Z.); 2 College of Biotechnology and Pharmaceutical Engineering, West Anhui University, Lu’an 237499, China; 02000179@wxc.edu.cn; 3 Key Laboratory of Marine Fishery Resources Exploitment and Utilization of Zhejiang Province, Zhejiang University of Technology, Hangzhou 310014, China

**Keywords:** marine actinomycetes, secondary metabolites, isolation

## Abstract

In this study, a detailed chemical investigation of a streptomycin-resistant strain of the deep-sea marine, actinomycete *Amycolatopsis* sp. WP1, yielded six novel amycolachromones A–F (**1**–**6**), together with five known analogues (**7**–**11**). Amycolachromones A–B (**1**–**2**) possessed unique dimer skeletons. The structures and relative configurations of compounds **1**–**11** were elucidated by extensive spectroscopic data analyses combined with X-ray crystal diffraction analysis. Plausible biogenetic pathways of amycolachromones A–F were also proposed.

## 1. Introduction

Marine microbial natural products, especially those derived from marine actinomycetes, have become an important source of novel bioactive compounds [1,2,3]. However, traditional screening strategies generally do not provide access to the full array of secondary metabolites encoded within actinomycete genomes [4]. For example, *Streptomyces coelicolor* initially produces four classes of metabolites using laboratory fermentation, despite genome sequencing revealing the capacity to produce >30 families of metabolites [5,6]. To solve this problem, various strategies have been proposed to activate the expression of otherwise silent biosynthetic gene clusters, including the ‘one strain many compounds’ (OSMAC) approach [7], co-cultivation with other microorganisms [8] and chemical epigenetics [9]. Recently, a ribosome engineering approach that targets ribosomal proteins or RNA polymerase (RNAP) has shown promise for expression of cryptic gene clusters. This method selects for mutants that are resistant to antibiotics that target the bacterial ribosome, presumably activating the expression of bacterial cryptic genes by resistant mutants [10,11]. Shima and co-workers demonstrated this method in actinomycetes by activating the biosynthetic pathway for actinorhodin in mutant Streptomyces that developed resistance to streptomycin [12]. Recent adoptions of this approach demonstrated the ability of streptomycin-resistant mutants to enhance production of actinolactomycin [13], fredericamycin A and chlorinated alkaloids, inducamides A–C [14,15].

**Figure 1:**
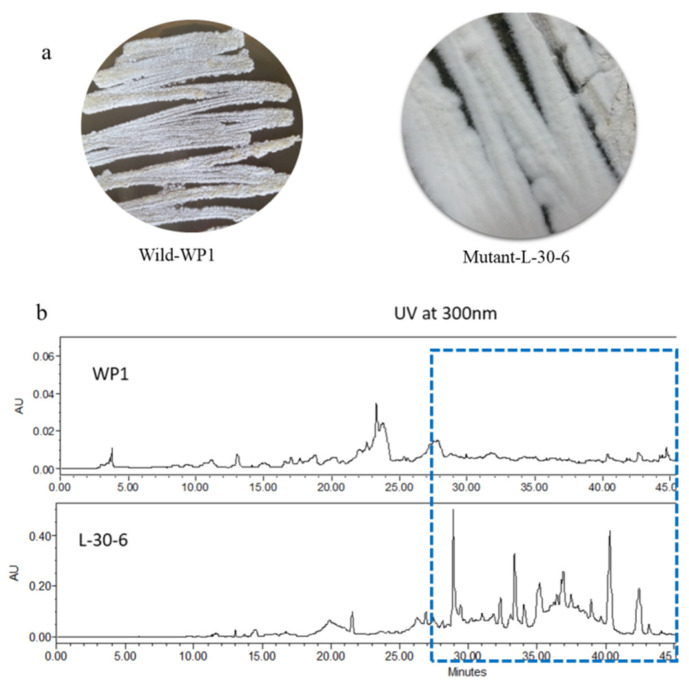
(**a**) Wild-type strain WP1 and streptomycin-resistant strain l-30-6 grown under identical conditions on ISP_2_ media. (**b**) HPLC traces of wt-WP1 and mutant l-30-6 showing the production of new compounds (UV detection at 300 nm).

**Figure 2:**
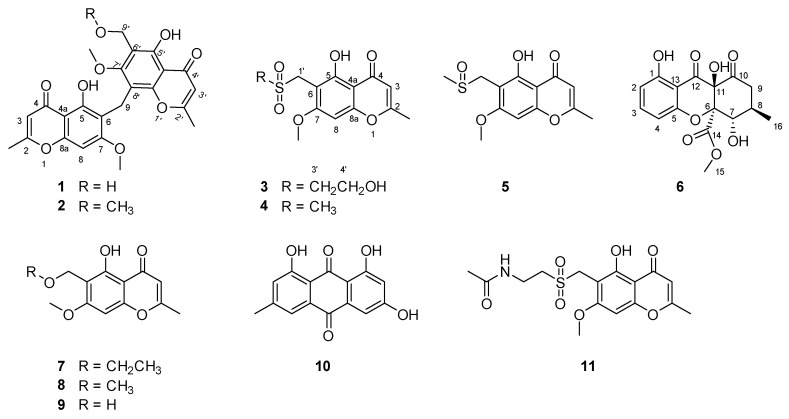
Chemical structures of compounds **1**–**11**.

Chromones are oxygen-containing heterocyclic compounds with a chromone benzoannelated γ-pyrone ring (4H-chromen-4-one, 4H-1-benzopyran-4-one) that are widely distributed in bacteria, fungi and plant [16]. Chromones and analogues can be considered privileged structures in drug discovery due to their numerous biological activities, such as anti-inflammatory, antiplatelet, anticancer, antimicrobial, anti-neurodegenerative and anti-obesity effects [17]. In this paper, we undertook a ribosome engineering approach for activating biosynthetic pathways in *Amycolatopsis* sp. WP1, a deep sea actinomycete isolated from sediments collected at −2945 m in the Indian Ocean. A streptomycin-resistant strain, designated as L-30-6 (Figure 1), was observed to produce six new chromone derivatives, designated as the amycolachromones A–F (**1**–**6**), and five known chromone derivatives (**7**–**11**) (Figure 2).

## 2. Results and Discussion

Amycolachromone A (**1**) displayed HRESIMS peak at *m*/*z* 477.1172 [M + Na]^+^ (calcd 477.1162) corresponding to the molecular formula C_24_H_22_O_9_, indicating fourteen degrees of unsaturation. Analysis of the NMR data of **1** (Table 1, see Supplementary Materials) revealed three aromatic protons at *δ*_H_ 6.64 (1H, s, H-8), 6.22 (1H, s, H-3′), 6.20 (1H, s, H-3), two methoxy groups at *δ*_H_ 3.83 (3H, s, CH_3_O-7) and 3.75 (3H, s, CH_3_O-7′), two methyl groups at *δ*_H_ 2.35 (3H, s, CH_3_-2) and 3.21 (3H, s, CH_3_-2′), two methylenes at *δ*_H_ 4.45 (2H, d, *J* = 4.8 Hz, H-9′) and 3.98 (2H, s, H-9), two phenolic hydroxyl groups at *δ*_H_ 13.13 (1H s, OH-5) and 13.10 (1H s, OH-5′), a hydroxyl group at *δ*_H_ 4.78 (1H, t, *J* = 5.2 Hz, OH-9′). The ^13^C NMR (Table 1) revealed 24 carbon signals: the two carbonyls C-4 (*δ*_C_ 183.2) and C-4′ (*δ*_C_ 182.5), the three aromatic carbons C-3 (*δ*_C_ 108.7), C-3′ (*δ*_C_ 108.4) and C-8 (*δ*_C_ 90.6), five non-oxygenated quaternary aromatic carbons at C-4a (*δ*_C_ 110.8), C-6 (*δ*_C_ 104.4), C-4a’ (*δ*_C_ 106.7), C-6′ (*δ*_C_ 117.6), and C-8′ (*δ*_C_ 112.5), eight oxygenated quaternary aromatic carbons at C-2 (*δ*_C_ 168.5), C-2′ (*δ*_C_ 168.3), C-7 (*δ*_C_ 163.8),C-7′ (*δ*_C_ 163.5), C-5 (*δ*_C_ 158.5), C-5′ (*δ*_C_ 158.3), C-8a (*δ*_C_ 156.7), and C-8a’ (*δ*_C_ 154.8), two methoxy groups CH_3_O-7 (*δ*_C_ 63.2) and CH_3_O-7′ (*δ*_C_ 56.7), two methyl groups CH_3_-2 (*δ*_C_ 20.4) and CH_3_-2′ (*δ*_C_ 20.0), and two methylenes C-9′ (*δ*_C_ 52.1) and C-9 (*δ*_C_ 16.9). Analysis of the ^1^H and ^13^C NMR data of **1** revealed the presence of the same 5-hydroxy-4H-chromen-4-one moiety as found in xanthones [18,19], and therefore suggested a compound comprising two xanthone building blocks.

**Figure 3:**
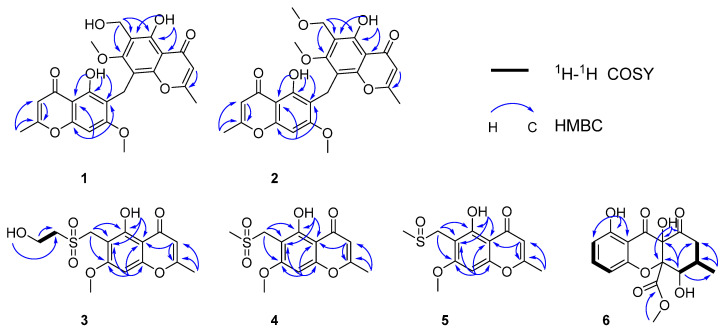
Key HMBC and COSY correlations of compounds **1**–**6**.

^1^H-^1^H COSY correlations were observed from H-9′ to OH-9′. Further confirmation was found for HMBC correlations of 5-OH to C-5, C-6, 4a; H-8 to C-6, C-4a, C-8a; H-3 to C-2; 2-CH_3_ to C-2, C-3, indicating the same Eugenin. HMBC correlations from 5′-OH to C-5′, C-6′, 4a′; H-3′ to C-2′; 2′-CH_3_ to C-2′, C-3′; H-9′ to C-5′, C-6′, C-7′, indicated the same 6-Hydroxymethyleugenin (**10**) [20]. HMBC correlations from H-9 to C-7, C-5, C-4a, C8′, C-7, C-8a′, indicated that Eugenin and 6-hydroxymethyleugenin are linked with C-9. Selected key correlations in the observed NMR spectrum are shown in Figure 3. On the basis of these results, the structure of compound **1** was established as shown.

Amycolachromone B (**2**) displayed HRESIMS peak at *m*/*z* 469.1502 [M + H]^+^ (calcd 469.1499), *m*/*z* 491.1333 [M + Na]^+^ (calcd 469.1318), corresponding to the molecular formula C_25_H_24_O_9_ (fourteen degrees of unsaturation). Analysis of the NMR data of **2** (Table 1) revealed for three aromatic protons at *δ*_H_ 6.64 (1H, s, H-8), 6.22 (1H, s, H-3′), 6.23 (1H, s, H-3), two methoxy groups at *δ*_H_ 3.82 (3H, s, CH_3_O-7) and 3.76 (3H, s, CH_3_O-7′), two methyl groups at *δ*_H_ 2.22 (3H, s, CH_3_-2) and 3.37 (3H, s, CH_3_-2′), two methylene at *δ*_H_ 4.35 (2H, s, H-9′) and 3.98 (2H, s, H-9), two phenolic hydroxyl groups at *δ*_H_ 13.21 (1H s, OH-5) and 13.11 (1H s, OH-5′). The ^13^C NMR (Table 1) revealed 25 carbon signals: the two carbonyl group C-4 (*δ*_C_ 183.2), C-4′ (*δ*_C_ 182.4), three aromatic carbon C-3 (*δ*_C_ 108.7), C-3′ (*δ*_C_ 108.5) and C-8 (*δ*_C_ 90.6), five nonoxygenated quaternary aromatic carbons at C-4a (*δ*_C_ 110.8), C-6 (*δ*_C_ 100.9), C-4a′ (*δ*_C_ 106.7), C-6′ (*δ*_C_ 114.1), and C-8′ (*δ*_C_ 112.5), eight oxygenated quaternary aromatic carbons at C-2 (*δ*_C_ 168.8), C-2′ (*δ*_C_ 168.2), C-7 (*δ*_C_ 164.2), C-7′ (*δ*_C_ 163.4), C-5 (*δ*_C_ 158.9), C-5′ (*δ*_C_ 158.6),C-8a (*δ*_C_ 156.7), and C-8a′ (*δ*_C_ 154.8), three methoxy groups CH_3_O-7(*δ*_C_ 62.5), CH_3_O-9′ (*δ*_C_ 57.7), and CH_3_O-7′ (*δ*_C_ 56.6), two methyl groups CH_3_-2 (*δ*_C_ 20.4) and CH_3_-2′ (*δ*_C_ 20.0), two methylene C-9′ (*δ*_C_ 63.2) and C-9 (*δ*_C_ 16.9). Analysis of the ^1^H and ^13^C NMR data of **2** revealed the presence of the same 5-hydroxy-4H-chromen-4-one moiety as found in xanthones [18,19], and comprised two xanthones. In contrast, the NMR data of **2** showed them to be nearly identical except for a methoxy group linked with C-9′. Further confirmation was found for HMBC correlations of 5-OH to C-5, C-6, 4a; H-8 to C-6, C-4a, C-8a, C-7; H-3 to C-2; 2-CH_3_ to C-2, C-3, indicated that same as Eugenin [20]. HMBC correlations from 5′-OH to C-5′, C-6′, 4a′; H-3′ to C-2′; 2′-CH_3_ to C-2′, C-3′; H-9′ to C-5′, C-6′, C-7′, indicated that same as 6-Methoxymethyleugenin (**9**) [21]. HMBC correlations from H-9 to C-7, C-5, C-4a, C8′, C-7, C-8a′, indicated that Eugenin and Methoxymethyleugenin are linked with C-9. Selected key correlations in the observed NMR spectrum are shown in Figure 3. On the basis of these results, the structure of compound **2** was established as shown.

Amycolachromone C (**3**) displayed HRESIMS ion at *m*/*z* 351.0515 [M + Na]^+^ (calcd 351.0514), corresponding to the molecular formula C_14_H_16_O_7_S, indicating nine degrees of unsaturation. Analysis of the NMR data of **3** (Table 2) revealed two aromatic protons at *δ*_H_ 6.78 (1H, s, H-8), 6.32 (1H, s, H-3), a methoxy group at *δ*_H_ 3.90 (3H, s, CH_3_O-7), a methyl group at *δ*_H_ 2.39 (3H, s, CH_3_-2), three methylenes at *δ*_H_ 4.39 (2H, s, H-1′), *δ*_H_ 3.80 (2H, q, *J* =6.1 Hz, H-4′), and 3.21 (2H, t, *J* = 6.3, H-3′), a phenolic hydroxyl group at *δ*_H_ 11.40 (1H s, OH-5), and a hydroxyl group at *δ*_H_ 5.03 (1H, t, *J* = 5.4 Hz, 1H, OH-4′). Examination of the ^13^C NMR spectrum (Table 2) revealed 14 carbon signals: a carbonyl group C-4 (*δ*_C_ 182.4), the two aromatic carbons C-3 (*δ*_C_ 108.9) and C-8 (*δ*_C_ 91.3), three non-oxygenated quaternary aromatic carbons at C-8a (*δ*_C_ 158.2), C-4a (*δ*_C_ 104.6), and C-6 (*δ*_C_ 101.0), and three oxygenated quaternary aromatic carbons at C-2 (*δ*_C_ 168.9), C-5 (*δ*_C_ 159.9), and C-7 (*δ*_C_ 163.9) and the methylene C-1′ (*δ*_C_ 49.3). Analysis of the ^1^H and ^13^C NMR data of **2** revealed the presence of the same 5-hydroxy-4H-chromen-4-one moiety as found in xanthones [18,19].

In the ^1^H-^1^H COSY spectrum, there were correlations from H-4′ to OH-5′ and H-3′. According to the HMBC, there were correlations from H-1′ to C-6, C-5, and C-7, H-4′ to C-3′, OH-4′ to C-3′. The sulfur atom present in **3** was shown to be attached at C-1′and C-3′, indicated that C-1′was attached at C-6. Further confirmation was found for HMBC correlations of OH-5 to C-4a, C-6, C-5; CH_3_-2 to C-3, C-2; CH_3_O-7 to C7, H-3 to CH_3_-2, C-4a, C-2; H-8 to C-4a, C-6, C-8a, C-7, C-4, a hydroxyl group could be located at C-5, a methoxy groups could be located at C-7, a methyl group could be located at C-2. Selected key correlations in the observed NMR spectrum are shown in Figure 3. On the basis of these results, the structure of compound **3** was established as shown.

Amycolachromone D (**4**) displayed HRESIMS peak at *m*/*z* 321.0406 [M + Na]^+^ (calcd 321.0409), corresponding to the molecular formula C_13_H_14_O_6_S (nine degrees of unsaturation). Analysis of the NMR data of **4** (Table 2) revealed for two aromatic protons at *δ*_H_ 6.76 (1H, s, H-8), 6.30 (1H, s, H-3), a methoxy groups at *δ*_H_ 3.90 (3H, s, CH_3_O-7), two methyl groups at *δ*_H_ 2.91 (3H, s, H-3′) and 2.39 (3H, s, CH_3_-2), a methylene group at *δ*_H_ 4.33 (2H, s, H-1′). The ^13^C NMR (Table 2) revealed 13 carbon signals: a carbonyl group C-4 (*δ*_C_ 182.3), two aromatic carbon C-3 (*δ*_C_ 108.9) and C-8 (*δ*_C_ 91.1), three nonoxygenated quaternary aromatic carbons at C-8a (*δ*_C_ 158.2),C-4a (*δ*_C_ 104.7), and C-6 (*δ*_C_ 101.2), three oxygenated quaternary aromatic carbons at C-2 (*δ*_C_ 168.7), C-5 (*δ*_C_ 159.1), and C-7 (*δ*_C_ 163.7), a methoxy groups CH_3_O-7 (*δ*_C_ 57.2), two methyl groups C-3′ (*δ*_C_ 42.0) and CH_3_-2 (*δ*_C_ 20.4), a methylene group C-1′ (*δ*_C_ 49.4). Analysis of the ^1^H and ^13^C NMR data of **4** revealed the presence of the same 5-hydroxy-4H-chromen-4-one moiety as found in xanthones [18,19]. A side-by-side comparison of the NMR spectroscopic data with those of **3** showed them to be nearly identical except for the final hydroxymethyl unit on the side chain.

According to the HMBC correlations from H-1′ to C-6, C-5 and C-7, the sulfur atom present in **4** was shown to be attached at C-1′and C-3′, indicating that C-1′ was attached at C-6, Further confirmation was found for HMBC correlations of CH_3_O-7 to C7; H-3 to CH_3_-2, C-4a, C-2; H-8 to C-4a, C-6, C-8a, C-7, C-4, a methoxy group could be located at C-7 and a methyl group could be located at C-2. Selected key correlations in the observed NMR spectrum are shown in Figure 3. On the basis of these results, the structure of compound **4** was established as shown.

Amycolachromone E (**5**) displayed HRESIMS peak at *m*/*z* 305.0462 [M + Na]^+^ (calcd 305.0460), corresponding to the molecular formula C_13_H_14_O_5_S (eight degrees of unsaturation). Analysis of the NMR data of **5** (Table 2) revealed for two aromatic protons at *δ*_H_ 6.76 (1H, s, H-8), 6.29 (1H, s, H-3), a methoxy groups at *δ*_H_ 3.90 (3H, s, CH_3_O-7), two methyl groups at *δ*_H_ 2.54 (3H, s, H-3′) and 2.39 (3H, s, CH_3_-2) and a methylene group at *δ*_H_ 3.99 (2H, d, *J* = 6.7 Hz, H-1′). The ^13^C NMR (Table 2) revealed 13 carbon signals: a carbonyl group C-4 (*δ*_C_ 182.3), two aromatic carbon C-3 (*δ*_C_ 108.9) and C-8 (*δ*_C_ 91.2), three nonoxygenated quaternary aromatic carbons at C-8a (*δ*_C_ 157.8), C-4a (*δ*_C_ 104.7), and C-6 (*δ*_C_ 102.3), three oxygenated quaternary aromatic carbons at C-2 (*δ*_C_ 168.8), C-5 (*δ*_C_ 159.6), and C-7 (*δ*_C_ 163.6), a methoxy groups CH_3_O-7 (*δ*_C_ 57.2), two methyl groups C-3′ (*δ*_C_ 39.1) and CH_3_-2 (*δ*_C_ 20.4), a methylene group C-1′ (*δ*_C_ 48.4). Analysis of the ^1^H and ^13^C NMR data of **5** revealed the presence of the same 5-hydroxy-4H-chromen-4-one moiety as found in xanthones [18,19]. A side-by-side comparison of the NMR spectroscopic data with those of **3** showed them to be nearly identical except for the final sulfur monoxide unit on the side chain.

According to the HMBC correlations from H-1′ to C-6, C-5 and C-7, H-3′ to C-1′, the sulfur atom present in **5** was shown to be attached at C-1′and C-3′, indicating that C-1′ was attached at C-6, Further confirmation was found for HMBC correlations of CH_3_O-7 to C7; H-3 to CH_3_-2, C-4a, C-2; H-8 to C-4a, C-6, C-8a, C-7 and C-4, a methoxy group could be located at C-7, a methyl group could be located at C-2. Selected key correlations in the observed NMR spectrum are shown in Figure 3. On the basis of these results, the structure of compound **5** was established as shown.

**Figure 4:**
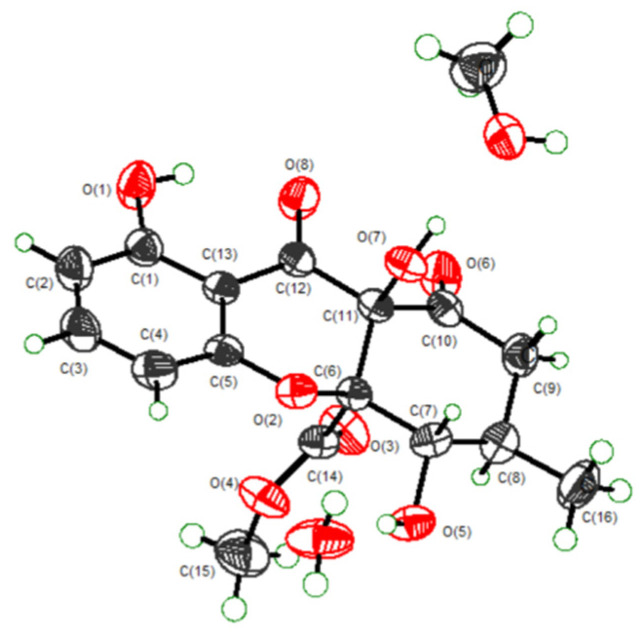
ORTEP diagram for the single-crystal X-ray of Amycochromone F (**6**).

Amycolachromone F (**6**), ${\left[\alpha \right]}_{D}^{25}$−54 (*c* 0.1, MeOH), displayed HRESIMS peak at *m*/*z* 337.0915 [M + H]^+^ (calcd 337.0923), corresponding to the molecular formula C_16_H_16_O_8_ (nine degrees of unsaturation). Analysis of the ^1^H data of **6** (Table 3) revealed resonance for three aromatic protons at *δ*_H_ 7.52 (1H, t, *J* = 8.3 Hz, H-3), 6.60 (1H, d, *J* = 8.3 Hz, H-4), 6.53 (1H, d, *J* = 8.3 Hz, H-2), a methoxy group at *δ*_H_ 3.50 (3H, s, H-15), a methyl group at *δ*_H_ 1.06 (3H, d, *J* = 6.4 Hz, H-16), a methylene at *δ*_H_ 2.81 (1H, dd, *J* = 14.4, 12.9 Hz, H-9a) and 2.25 (1H, dd, *J* = 14.5, 5.3 Hz, H-9b), a oxygenated methine at *δ*_H_ 4.20 (1H, dd, *J* = 10.5, 6.0 Hz, H-7), a methine at *δ*_H_ 1.97–1.86 (1H, m, H-8), three hydroxyl groups at *δ*_H_ 11.35 (1H s, OH-1), 8.09 (1H, s, OH-11), and 5.91 (1H, d, *J* = 6.0 Hz, OH-7). The ^13^C NMR (Table 3) revealed sixteen carbon signals: three carbonyl group C-10 (*δ*_C_ 198.6), C-12 (*δ*_C_ 191.8) and C-14 (*δ*_C_ 168.5), three aromatic carbon C-3 (*δ*_C_ 138.7), C-2 (*δ*_C_ 109.5), and C-4 (*δ*_C_ 107.4), a nonoxygenated quaternary aromatic carbons at C-13 (*δ*_C_ 106.5), two oxygenated quaternary aromatic carbons at C-1 (*δ*_C_ 161.9) and C-5 (*δ*_C_ 158.9), two sp^3^-quaternary carbon C-11 (*δ*_C_ 90.0) and C-6 (*δ*_C_ 73.0), a methoxy group C-15 (*δ*_C_ 52.7), a methyl group C-16 (*δ*_C_ 18.6), an oxygenated methine C-7 (*δ*_C_ 71.8), a methine C-8 (*δ*_C_ 31.1) and a methylene C-9 group (*δ*_C_ 43.1). Analysis of the ^1^H and ^13^C NMR data of **6** revealed the presence of the same 5-hydroxy-4H-chromen-4-one moiety as found in xanthones [18,19]. In the ^1^H-^1^H COSY spectrum, the correlations from H-7 to H-8 and OH-7, from H-8 to H-9 and H-16. Further confirmation was found for HMBC correlations of H-7 to C-16, C-6, C-11 and C-9; H_3_-16 to C-7, C-8 and C-9, indicated that C-16 was attached to C-8, and OH-7 was located at C-7. HMBC correlations from the O-methyl proton signal H_3_-15 to the carboxylic carbon C-14 confirmed that the O-methyl group was located at C-14. HMBC correlations from OH-11 to C-11, C-6 and C-10, OH-1 to C-2, C-13 and C-1 indicated that OH-1 and OH-11 were attached to C-1 and C-11, respectively [22,23]. Selected key correlations in the observed NMR spectrum are shown in Figure 3. Thus, the planar structure of **6** was established. Moreover, the relative configuration of **6** was established to be 6R*, 7S*, 8R* and 11R* by X-ray crystallography using Mo K*a* radiation (Figure 4).

**Scheme 1:**
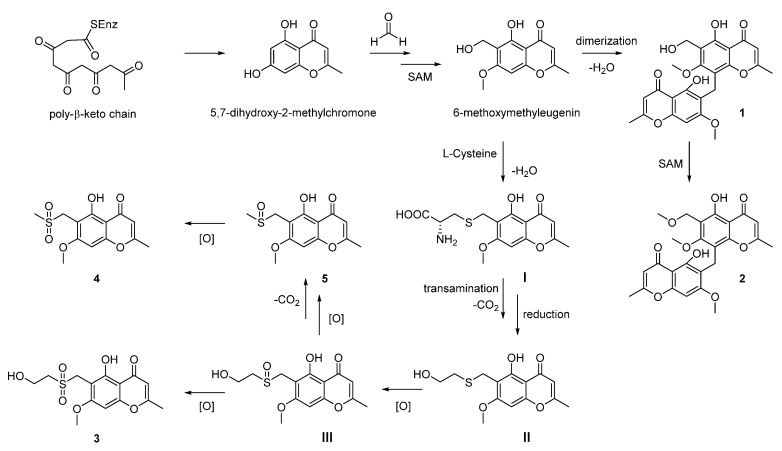
Proposed hypothetical biosynthesis pathway of **1**–**5**.

**Scheme 2:**
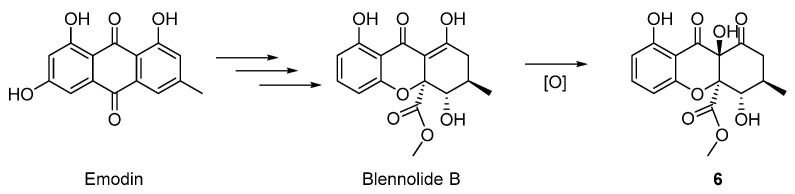
Proposed hypothetical biosynthesis pathway of **6**.

Further analysis of the structures allowed us to raise a plausible biosynthetic pathway of compounds **1**–**6**. As outlined in the Scheme 1, compounds **1**–**5** were structurally related to the known metabolite 6-methoxymethyleugenin, which was derived from the widely existing 5,7-dihydroxy-2-methylchromone via the hydroxymethylation with formaldehyde and the methylation with SAM (*S*-adenosyl methionine). The compound **1** was the dimerization of 6-methoxymethyleugenin, and the sequential methylation with SAM could afford the related compound **2**. For compound **3**–**5**, we proposed that the sulfur in these structures was from l-cysteine. Thus, the Michael addition of l-cysteine to the *ortho*-quinone methide intermediate from 6-methoxymethyleugenin gave the compound **I**. Then, transamination, decarboxylation and reduction sequence of **I** furnished the 2-sulfo-ethanol **II** occurred. An oxidation of sulfur in **II** gave the compound **III**. Finally, compound **3** was obtained through the double oxidation of sulfur in **II**. The oxidation of the hydroxyl group in **III** to the corresponding carboxylic acid occurred and followed with a decarboxylation afforded for compound **5**. Furthermore, compound **4** was the oxidation product of **5 [24,25]**. In addition, compound **6** was the oxidation product of the known natural product blennolide B, which was proposed by Franck to be a derivative of emodin (Scheme 2) [26].

The structures of five known compounds were identified as 6-ethoxymethyleugenin (**7**), 6-methoxymethyleugenin (**8**), 6-hydroxymethyleugenin (**9**), emodin (**10**) and the ascomycete metabolite chaetoquadrin D (**11**) by comparison of spectroscopic data with reported values and are described here for the first time as produced by *Amycolatopsis* sp.

The AlkB family of DNA repair enzymes utilize an α-ketoglutarate/Fe(II)-dependent mechanism to oxidize the aberrant alkyl groups, finally repairing alkyl DNA bases [27,28]. Compounds **1**–**11** were evaluated for their in vitro ABH2 inhibitory activities. Compounds **1**–**11** exhibited weak inhibitory activity against the ABH2 enzyme. However, in 2019, a paper was published that tested emodin (**10**). It exhibited strong inhibitory activity for the ALKH3 enzyme with IC_50_ of 8.8 μM [29]. This hinted that these compounds might inhibit other members of the AlkB family of enzymes. 

In conclusion, the chemical investigation of a streptomycin-resistant strain of the deep-sea marine actinomycete, *Amycolatopsis* sp. WP1, led to the isolation and identification of six novel compounds, amycolachromones A–F (**1**–**6**) and five known analogues (**7**–**11**). Among them, amycolachromones A–B (**1**–**2**) represents an unusual fused skeleton between two 6-hydroxymethyleugenin, and the relative configuration of amycolachromones F (**6**) was determined by the signal-crystal X-ray diffraction. The discovery of amycolachromones A–F not only expanded the chemical diversity of natural products and inspire further synthetic studies, but also provided a template for the exploration of inhibitors of other members of the AlkB family of enzymes.

## 3. Materials and Methods

General experimental procedures. All chemical reagents and solvents were purchased from Sigma–Aldrich (Shanghai, China). UV spectra were acquired with a DU 800 UV/vis spectrophotometer (Beckman Coulter, Brea, CA, USA). IR spectra were acquired with a Nicolet 380 FT-IR (Thermo Electron Corporation, Beverly, MA, USA). NMR experiments were conducted using an Agilent NMR 500 MHz spectrometer (Santa Clara, CA, USA) and BRUKER NMR 600 MHz spectrometer (San Jose, CA, USA) with (CD_3_)_2_SO as the solvent (referenced to residual DMSO at *δ*_H_ 2.54 and *δ*_C_ 39.5) at 25 °C. Electrospray ionization mass spectra (ESIMS) were acquired using an AB Sciex TripleTOF 4600 spectrometer (Boston, MA, USA) in the positive and negative ion mode. HPLC experiments were performed on a Hitachi Elite LaChrom system (Tokyo, Japan) equipped with a diode array detector model L-2450, pump L-2130 and autosampler L-2200. Semipreparative HPLC experiments were completed with a Waters XBridge Prep C_18_ (Miflord, CO, USA) 5 μm, 10 mm × 250 mm column and Phenomenex Luna C_18_ 5 μm, 250 mm × 21.2 mm column.

Bacterial Strain and Culture Conditions. The WP1 strain (CGMCC No. 10738) was isolated from deep-sea sediments of the Southwest Indian Ocean and identified as *Amycolatopsis* sp. by 16S rRNA sequence comparison. WP1 was grown in ISP_2_ medium consisting of 1.0% (*w*/*v*) malt extract, 0.4% (*w*/*v*) yeast extract, 0.4% (*w*/*v*) glucose and 3% (*w*/*v*) sea salt, the pH of medium was adjusted to 7.4 using 2 M HCl and 2 M NaOH.

Mutants of strain WP1. The WP1 strain suspensions were spread onto ISP_2_ plates containing different concentrations (0, 10, 20, 30, 40, 50 and 60 mg/mL) of streptomycin. The plates were incubated at 37 °C for 7 days. Mutant colonies producing the white pigment different than the WP1 strain were selected, generating mutant strain L-30-6, which was obtained on the IPS_2_ plate containing 30 mg/mL streptomycin.

Extraction and isolation. The mutant L-30-6 strain was inoculated into ISP_2_ broth with 3% sea salt in 250 mL Erlenmeyer flasks, at 30 °C on a rotary shaker at 180 rpm for 2 days as seed culture. Each of the seed cultures (32 mL) was transferred into 1 L Erlenmeyer flasks containing 400 mL of ISP_2_ supplemented with 3% sea salt. These flasks were incubated at 30 °C on a rotary shaker at 180 rpm for 6 days. The resulting cultures (60 L) were centrifuged to yield the supernatant and a mycelial pellet. The supernatant was adsorbed onto macroporous resin XAD16N (DOW, St. Louis, Missouri, CA, USA) and eluted with linear gradient of 0–100% EtOH in H_2_O to afford six fractions (A–F).

Fraction C (3.8 g) was subjected to semipreparative HPLC (Phenomenex Luna C_18_, 250 mm × 21.2 mm, 5 µm, 10 mL/min) using a gradient solvent from 40–90% MeOH in H_2_O over 30 min to give five fractions (C1–C5). Fraction C2 was further purified by semipreparative HPLC (Waters XBridge Prep C_18_ 5 μm, 10 mm× 250 mm, 4 mL/min) using an isocratic solvent system of CH_3_CN:H_2_O (15:85) over 30 min to afford compound **6** (10.2 mg) and C2A. Subfraction C2A was further purified by preparative HPLC with MeOH-H_2_O (45:55) to provide compound **7** (2.6 mg). Fraction C3 was further purified by semipreparative HPLC with MeOH-H_2_O (45:55) to yield compound **12** (6.5 mg), **3** (3.1 mg) and **4** (2.2 mg). Fraction C4 was further purified by semipreparative HPLC with MeOH:H_2_O (45:55) to afford compound **5** (2.2 mg).

Fraction D (2.1 g) was subjected to semipreparative HPLC (Phenomenex Luna C18, 250 mm × 21.2 mm, 5 µm,10 mL/min) using a gradient solvent from 50–80% MeOH in H_2_O over 30 min to generate five fractions (D1–D5). Fraction D3 was further purified by semipreparative HPLC using an isocratic solvent system of MeCN:H_2_O (50:50) to afford compound **1** (1.7 mg) and compound **2** (1.8 mg). D4 was subjected to preparative HPLC with MeCN:H_2_O (30:70) to provide compounds **9** (35.3 mg) and **10** (13.5 mg). D5 was further purified by preparative HPLC with MeOH-H_2_O (45:55) to yield compounds **8** (1.9 mg) and **11** (6.8 mg). The following are details of the extraction and isolation of the compounds.

Amycolachromone A (**1**): White, amorphous powder; UV (MeOH) λmax (log ε) 253 (3.28) nm; IR (ZnSe) *ν*_max_ 3426, 3195, 2844, 1656, 1445, 1008 cm^−1^; ^1^H and ^13^C NMR data, Table 1; HRESIMS *m*/*z* 477.1172 [M + Na]^+^ (calcd for C_24_H_22_O_9_, 477.1162).

Amycolachromone B (**2**): White, amorphous powder; UV (MeOH) λmax (log ε) 254 (3.46) nm; IR (ZnSe) *ν*_max_ 3460, 3190, 2894, 1658, 1445, 1008 cm^−1^; ^1^H and ^13^C NMR data, Table 1; HRESIMS *m*/*z* 469.1502 [M + H]^+^ (calcd for C_25_H_24_O_9_, 469.1499), *m*/*z* 491.1333 [M + Na]^+^ (calcd 469.1318).

Amycolachromone C (**3**): White, amorphous powder; UV (MeOH) λmax (log ε) 250 (3.43), 233 (3.46) nm; IR (ZnSe) *ν*_max_ 3420, 3199, 2993, 1650, 1310, 1089 cm^−1^; ^1^H and ^13^C NMR data, Table 2; HRESIMS *m*/*z* 351.0515 [M + Na]^+^ (calcd for C_14_H_16_O_7_S, 351.0514).

Amycolachromone D (**4**): White, amorphous powder; UV (MeOH) λmax (log ε) 250 (3.56), 233 (3.58) nm; IR (ZnSe) *ν*_max_ 3520, 32,469, 2990, 1750, 1281, 1008 cm^−1^; ^1^H and ^13^C NMR data, Table 2; HRESIMS *m*/*z* 321.0406 [M + Na]^+^ (calcd for C_13_H_14_O_6_S, 321.0409).

Amycolachromone E (**5**): White, amorphous powder; UV (MeOH) λmax (log ε) 250 (3.61), 240 (3.61) nm; IR (ZnSe) *ν*_max_ 3470, 3122, 2880, 1603, 1210, 1089 cm^−1^; ^1^H and ^13^C NMR data, Table 2; HRESIMS *m*/*z* 305.0462 [M + Na]^+^ (calcd for C_13_H_14_O_5_S, 305.0460).

Amycolachromone F (**6**): White, crystal; UV (MeOH) λmax (log ε) 356 (3.20), 277 (3.68) nm; IR (ZnSe) *ν*_max_ 3477, 2956, 2916, 1748, 1622, 1475, 1349, 1083 cm^−1^; ^1^H and ^13^C NMR data, Table 3; HRESIMS *m*/*z* 337.0915 [M + H]^+^ (calcd for C_16_H_16_O_8_, 337.0923).

6-Ethoxymethyleugenin (**7**): White, amorphous powder; HR-ESIMS *m*/*z* 287.0891 [M + Na]^+^ (calcd for C_14_H_16_O_5_, 287.0985). ^1^H-NMR (600 MHz, DMSO-*d*_6_): *δ*_H_ 13.19 (s, OH-5), 6.70 (s, 1H, H-8), 6.28 (s, 1H, H-3), 4.41 (s, 2H, CH_2_OCH_3_), 3.89 (s, 3H, OCH_3_), 3.44 (q, *J* = 7.0 Hz, 2H, 6-CH_2_OC*H_2_*), 2.40 (s, 3H, 2-CH_3_), 1.08 (t, *J* = 7.0 Hz, 3H, OCH_2_C*H_3_*). ^13^C-NMR (150 MHz, DMSO-*d*_6_): *δ*_C_182.6 (C-4), 168.5 (C-2), 164.3 (C-7), 159.9 (C-5), 158.0 (C-8a), 109.1 (C-3), 108.9 (C-6), 104.4 (C-4a), 89.6 (C-8), 65.1 (*C*H_2_OCH_3_), 59.3 (6-CH_2_OCH_2_), 56.9 (7-OCH_3_), 20.4 (2-CH_3_) and 15.3 (CH_2_*C*H_3_).

6-Methoxymethyleugenin (**8**): White, amorphous powder; HR-ESIMS *m*/*z* 273.0738 [M + Na]^+^ (calcd for C_13_H_14_O, 273.0739). ^1^H-NMR (600 MHz, CDCl_3_): *δ*_H_ 13.04 (s, OH-5), 6.36 (s, 1H, H-8), 6.04 (s, 1H, H-3), 4.55 (s, 2H, C*H_2_*OCH_3_), 3.90 (s, 3H, OCH_3_), 3.40 (s, 3H, CH_2_OC*H_3_*), 2.35 (s, 3H, CH_3_). ^13^C-NMR (150 MHz, CDCl_3_): *δ*_C_182.4 (C-4), 166.6 (C-2), 164.2 (C-7), 160.6 (C-5), 158.2 (C-8a), 109.1 (C-3), 108.9 (C-6), 105.1 (C-4a), 89.6 (C-8), 61.6 (CH_2_OCH_3_), 58.2 (CH_2_OCH_3_), 56.2 (OCH_3_) and 20.4 (2-CH_3_).

6-Hydroxymethyleugenin (**9**): White, amorphous powder; HRESIMS *m*/*z* 259.0579 [M + Na]^+^ (calcd for C_12_H_12_O_5_, 259.0582). ^1^H-NMR (600 MHz, DMSO-*d*_6_): *δ*_H_ 13.09 (s, OH-5), 6.65 (s, 1H, H-8), 6.24 (s, 1H, H-3), 4.55 (t, *J* = 5.3 Hz, CH_2_O*H*), 4.43 (d, *J* = 5.2 Hz, 2H, H-9), 3.87 (s, 3H, OCH_3_), 2.38 (s, 3H, CH_3_). ^13^C-NMR (150 MHz, DMSO-*d*_6_): *δ*_C_182.5 (C-4), 168.4 (C-2), 164.1 (C-7), 159.1 (C-5), 157.7 (C-8a), 112.5 (C-6), 08.8 (C-3), 104.6 (C-4a), 90.7 (C-8), 56.7 (OCH_3_), 50.9 (CH_2_OH) and 20.4(CH_3_).

Emodin (**10**): White, amorphous powder; HRESIMS *m*/*z* 269.0448 [M − H]^−^ (calcd for C_15_H_12_O_5_, 269.0450). ^1^H-NMR (500 MHz, DMSO-*d*_6_): *δ*_H_ 12.07 (d, *J* = 19.5 Hz, 2H, OH), 7.49 (d, *J* = 1.1 Hz, 1H, H-5), 7.16 (s, 1H, H-7), 7.10 (d, *J* = 2.4 Hz, 1H, H-4), 6.57 (d, *J* = 2.4 Hz, 1H, H-2), 2.41 (s, 3H, CH_3_). ^13^C-NMR (150 MHz, DMSO-*d*_6_): *δ*_C_190.0 (C-9), 182.0 (C-10), 166.6 (C-6), 165.0 (C-8), 161.9 (C-1), 148.6 (C-3), 135.6 (C-10a), 133.3 (C-4a), 24.6 (C-2), 120.9 (C-4), 113.9 (C-9a), 109.6 (C-5), 109.2 (C-7), 108.4 (C-8a) and 22.0 (CH_3_).

Chaetoquadrin D (**11**): White, amorphous powder; HRESIMS *m*/*z* HR-ESIMS *m*/*z* 370.0969 [M + H]^+^ (calcd for C_16_H_19_NO_7_S, 370.0960). ^1^H-NMR (500 MHz, DMSO-*d*_6_): *δ*_H_ 13.43 (s, 5-OH), 8.08 (t, N*H*), 6.79 (s, H-8), 6.32 (s, H-3), 4.36 (s, H-1′), 3.92 (s, 7-OCH_3_), 3.45 (dd, *J* = 13.6, 6.1 Hz, H-4′), 3.20 (t, *J* = 7.0 Hz, H-3′), 2.41 (s, 2-CH_3_), 1.81 (s, H-7′). ^13^C-NMR (125 MHz, DMSO-*d*_6_): *δ*_C_ 182.4 (C-4), 70.1 (C-6′), 169.0 (C-2), 163.7 (C-7), 159.9 (C-5), 158.2 (C-8a), 108.9 (C-3), 104.5 (C-4a), 100.8 (C-6), 91.4 (C-8), 57.3 (7-OCH_3_), 52.9 (C-3′), 48.5 (C-1′), 32.9 (C-4′), 22.9 (C-7′) and 20.42 (2-CH_3_).

X-ray Crystallographic Analysis of Compound **6**. Crystals of **6** were obtained in the mixed solvent comprising MeOH and H_2_O, and crystallographic data were deposited at the Cambridge Crystallographic Data Centre (CCDC) under the reference number CCDC 1873441. The X-ray diffraction data were collected with Mo Kα radiation (λ = 0.71073 Å). The structure was solved by direct methods using the SHELXS-97 program. Orthorhombic C_16_H_16_O_8_, CH_4_O, H_2_O, a = 7.7760(3) Å, b = 8.6993(4) Å, c = 26.8196(11) Å, α = 90°, β = 90°, γ = 90°, V = 1814.23(13) Å3, Z = 4, ρCalcd = 1.414 g/cm^3^, μ = 0.118 mm^−1^, and F (0 0 0) = 816. Measurements were in the range 3.038° ≤ θ ≤ 26.368°, with 3697 independent reflections, of which 3133 unique reflections with I > 2σ (I) were collected for the analysis, Rint = 0.0332. The final R indices: R1 = 0.0455, wR2 = 0.1177 [I > 2σ(I)], R indices (all data): R1 = 0.0566, wR2 = 0.1256, and largest difference peak and hole: 0.560 and-0.231 e Å-3.

The ABH2 family DNA repair enzyme assay. Effects of compounds **1**–**11** on the ABH2 family demethylase activity reactions on m3c-ss-DNA were evaluated. All reactions were performed at 37 °C in reaction buffer [5 μM Fe(NH_4_)_2_(SO_4_)_2_, 0.93 mMα-ketoglutarate, 1.86 mM ascorbic acid, and 46.56 mM HEPES (pH 8.0)] for 1 h. Varying concentrations of compounds **1**–**11** (0, 5.0, 7.5, 20, 30, 40, 50, 75 and 100 μM) were used for tests. The m3c-ss-DNA was pre-mixed with reaction buffer in a concentration of 5.0 μM. The reactions were initiated by adding 2.0 μM ABH2. The reactions were stopped by adding 10.0 mM EDTA followed by heating to 95 °C for 5 min. All the results of reaction were analyzed by HPLC. All reaction samples were quantified by DNApac PA-100 column (4 mm × 250 mm, Thermo Scientific, (Waltham, MA, USA) with isocratic 60 % mobile B, 1.5 M ammonium acetate, under a constant flow rate of 1.0 mL/min. Mobile A was water. The UV detection wavelength was 260 nm.

## Figures and Tables

**Table 1:** ^1^H (500 MHz) and ^13^C (125 MHz) NMR data of compounds **1** and **2** in DMSO-*d_6_*.

Position	1		2	
	*δ*_H_	*δ*_C_, Type	*δ*_H_	*δ*_C_, Type
2		168.5, C		168.8, C
3	6.22 (s, 1H)	108.7, CH	6.23 (s, 1H)	108.7, CH
4		183.2, C		183.2, C
4a		110.8, C		110.8, C
5		158.5, C		158.9, C
6		104.4, C		100.9, C
7		163.8, C		164.2, C
8	6.64 (s, 1H)	90.6, CH	6.63 (s, 1H)	90.6, CH
8a		156.7, C		156.7, C
9	3.98 (s, 2H)	16.9, CH_2_	3.98 (s, 2H)	16.9, CH_2_
2′		168.3, C		168.2, C
3′	6.20 (s, 1H)	108.4, CH	6.22 (s, 1H)	108.5, CH
4′		182.5, C		182.4, C
4a′		106.7, C		106.7, C
5′		158.3, C		158.6, C
6′		117.6, C		114.1, C
7′		163.5, C		163.4, C
8′		112.2, C		112.5, C
8a′		154.8, C		154.8, C
9′	4.45 (d, *J* = 4.8 Hz, 2H)	52.1, CH_2_	4.35 (s, 2H)	63.2, CH_2_
2-CH_3_	2.35 (s, 3H)	20.4, CH_3_	2.35 (s, 3H)	20.4, CH_3_
7-OCH_3_	3.83 (s, 3H)	63.2, CH3	3.82 (s, 3H)	62.5, CH3
2′-CH_3_	2.21 (s, 3H)	20.0, CH_3_	2.21 (s, 3H)	20.0, CH_3_
7′-OCH_3_	3.75 (s, 3H)	56.7, CH_3_	3.76 (s, 3H)	56.6, CH_3_
9′-OCH_3_			3.29 (s, 3H)	57.7, CH_3_
5-OH	13.13 (s, 1H)		13.21 (s, 1H)	
5′-OH	13.10 (s, 1H)		13.11 (s, 1H)	
9′-OH	4.78 (t, *J* = 5.2 Hz, 1H)			

**Table 2:** ^1^H (500 MHz) and ^13^C (125 MHz) NMR data of compounds **3**–**5** in DMSO-*d*_6_.

Position	3		4		5	
	*δ*_H_	*δ*_C_, Type	*δ*_H_	*δ*_C_, Type	*δ*_H_	*δ*_C_, Type
2		168.9, qC		168.7, qC		168.8, qC
3	6.31, s	108.9, CH	6.30, s	108.9, CH	6.29, s	108.9, CH
4		182.4, qC		182.3, qC		182.3, qC
4a		104.6, qC		104.7, qC		104.7, qC
5		159.9, qC		159.1, qC		159.6, qC
6		101.0, qC		101.2, qC		102.3, qC
7		163.9, qC		163.7, qC		163.6, qC
8	6.78, s	91.3, CH	6.76, s	91.1, CH	6.76, s	91.2, CH
8a		158.2, qC		158.2, qC		157.8, qC
1′	4.39, s	49.3, CH_2_	4.33, s	49.4, CH_2_	3.99, d, (6.7)	48.4, CH_2_
3′	3.21, t (6.3)	56.6, CH_2_	2.91, s	42.0, CH_3_	2.54, s	39.1, CH_3_
4′	3.80, q (6.1)	55.3, CH_2_				
OH-5	13.40, s					
OH-4′	5.03, t (5.4)					
OCH_3_-7	3.90, s	57.2, CH_3_	3.90, s	57.2, CH_3_	3.90, s	57.2, CH_3_
CH_3_-2	2.39, s	20.4, CH_3_	2.39, s	20.4, CH_3_	2.39, s	20.4, CH_3_

**Table 3:** ^1^H (500 MHz) and ^13^C (125 MHz) NMR data of compound **6** in DMSO-*d_6_*.

Position	6	
	*δ*_H_	*δ*_C_, Type
1		161.9, qC
2	6.53, d (8.3)	109.5, CH
3	7.52, t (8.3)	138.7, CH
4	6.60, d (8.3)	107.4, CH
5		158.9, qC
6		73.0, qC
7	4.20, dd (10.5, 6.0)	71.8, CH
8	1.97–1.86, m	31.1, CH
9	2.81, dd (14.4, 12.9); 2.25 dd(14.5, 5.3)	43.1, CH_2_
10		198.6, qC
11		90.0, qC
12		191.8, qC
13		106.5, qC
14		168.5, qC
15	3.50, s	52.7, CH_3_
16	1.06, d (6.4)	18.6, CH_3_
OH-1	11.35, s	
OH-7	5.91, d (6.0)	
OH-11	8.09, s	

## Supplementary Materials

The following supporting information can be downloaded at: https://www.mdpi.com/article/10.3390/md20030162/s1. Figures S1–S35: 1D and 2D NMR, HRESI mass spectra, and crystal data for compounds **1**–**6**. Table S1. Crystallographic data for Amycochromone F (**6**).
