# Supplementary material for: Amycolachromones A–F, Isolated from a Streptomycin-Resistant Strain of the Deep-Sea Marine Actinomycete Amycolatopsis sp. WP1
Source: Mar Drugs. 2022 Feb 24;20(3):162. doi: 10.3390/md20030162 (PMC8949813; doi:10.3390/md20030162)

checkCIF/PLATON report

You have not supplied any structure factors. As a result the full set of tests cannot be run.

THIS REPORT IS FOR GUIDANCE ONLY. IF USED AS PART OF A REVIEW PROCEDURE FOR PUBLICATION, IT SHOULD NOT REPLACE THE EXPERTISE OF AN EXPERIENCED CRYSTALLOGRAPHIC REFEREE.

No syntax errors found. CIF dictionary Interpreting this report

Datablock: exp_phem_6b1

```
Bond precision:   C-C = 0.0047 A          Wavelength=0.71073

Cell:              a=7.7760(3)      b=8.6993(4)      c=26.8196(11)
                   alpha=90         beta=90          gamma=90
Temperature:       296 K

                Calculated                   Reported
Volume          1814.23(13)                  1814.23(13)
Space group     P 21 21 21                   P 21 21 21
Hall group      P 2ac 2ab                    P 2ac 2ab
Moiety formula  C16 H16 O8, C H4 O, H2 O     C16 H16 O8, C H4 O, H2 O
Sum formula     C17 H22 O10                  C17 H22 O10
Mr              386.35                       386.34
Dx,g cm-3       1.414                        1.414
Z               4                            4
Mu (mm-1)       0.118                        0.118
F000            816.0                        816.0
F000'           816.57
h,k,lmax        9,10,33                      9,10,33
Nref            3712[ 2153]                  3697
Tmin,Tmax       0.999,1.000                  0.669,1.000
Tmin'           0.999

Correction method= # Reported T Limits: Tmin=0.669 Tmax=1.000
AbsCorr = MULTI-SCAN

Data completeness= 1.72/1.00       Theta(max)= 26.368

R(reflections)= 0.0455( 3133)                    wR2(reflections)=
                                                 0.1256( 3697)
S = 1.011                   Npar= 255
```

The following ALERTS were generated. Each ALERT has the format
test-name_ALERT_alert-type_alert-level.
Click on the hyperlinks for more details of the test.

Alert level B

```
PLAT031_ALERT_4_B Refined Extinction Parameter Within Range of ...      2.000 Sigma
PLAT035_ALERT_1_B _chemical_absolute_configuration Info  Not Given     Please Do !
```

Alert level C

```
STRVA01_ALERT_4_C           Flack parameter is too small
           From the CIF: _refine_ls_abs_structure_Flack   -0.300
           From the CIF: _refine_ls_abs_structure_Flack_su    0.400
PLAT094_ALERT_2_C Ratio of Maximum / Minimum Residual Density ....       2.42 Report
PLAT340_ALERT_3_C Low Bond Precision on  C-C Bonds ...............    0.00475 Ang.
```

Alert level G

```
PLAT007_ALERT_5_G Number of Unrefined Donor-H Atoms ..............          6 Report
PLAT012_ALERT_1_G No       _shelx_res_checksum Found in CIF ......     Please Check
PLAT032_ALERT_4_G Std. Uncertainty on Flack Parameter Value High .      0.400 Report
PLAT432_ALERT_2_G Short Inter X...Y Contact  C00C     ..C00Q     .       3.15 Ang.
                                                  1+x,y,z  =      1_655 Check
PLAT720_ALERT_4_G Number of Unusual/Non-Standard Labels ..........         49 Note
PLAT791_ALERT_4_G Model has Chirality at C00D        (Sohnke SpGr)          R Verify
PLAT791_ALERT_4_G Model has Chirality at C00E        (Sohnke SpGr)          R Verify
PLAT791_ALERT_4_G Model has Chirality at C00I        (Sohnke SpGr)          S Verify
PLAT791_ALERT_4_G Model has Chirality at C00N        (Sohnke SpGr)          R Verify
PLAT933_ALERT_2_G Number of HKL-OMIT Records in Embedded .res File         12 Note
```

0 **ALERT level A** = Most likely a serious problem - resolve or explain
2 **ALERT level B** = A potentially serious problem, consider carefully
3 **ALERT level C** = Check. Ensure it is not caused by an omission or oversight
10 **ALERT level G** = General information/check it is not something unexpected

2 ALERT type 1 CIF construction/syntax error, inconsistent or missing data
3 ALERT type 2 Indicator that the structure model may be wrong or deficient
1 ALERT type 3 Indicator that the structure quality may be low
8 ALERT type 4 Improvement, methodology, query or suggestion
1 ALERT type 5 Informative message, check

It is advisable to attempt to resolve as many as possible of the alerts in all categories. Often the minor alerts point to easily fixed oversights, errors and omissions in your CIF or refinement strategy, so attention to these fine details can be worthwhile. In order to resolve some of the more serious problems it may be necessary to carry out additional measurements or structure refinements. However, the purpose of your study may justify the reported deviations and the more serious of these should normally be commented upon in the discussion or experimental section of a paper or in the "special_details" fields of the CIF. checkCIF was carefully designed to identify outliers and unusual parameters, but every test has its limitations and alerts that are not important in a particular case may appear. Conversely, the absence of alerts does not guarantee there are no aspects of the results needing attention. It is up to the individual to critically assess their own results and, if necessary, seek expert advice.

Publication of your CIF in IUCr journals

A basic structural check has been run on your CIF. These basic checks will be run on all CIFs submitted for publication in IUCr journals (*Acta Crystallographica*, *Journal of Applied Crystallography*, *Journal of Synchrotron Radiation*); however, if you intend to submit to *Acta Crystallographica Section C* or *E* or *IUCrData*, you should make sure that full publication checks are run on the final version of your CIF prior to submission.

Publication of your CIF in other journals

Please refer to the *Notes for Authors* of the relevant journal for any special instructions relating to CIF submission.

PLATON version of 20/01/2022; check.def file version of 19/01/2022

Datablock exp_phem_6b1 - ellipsoid plot

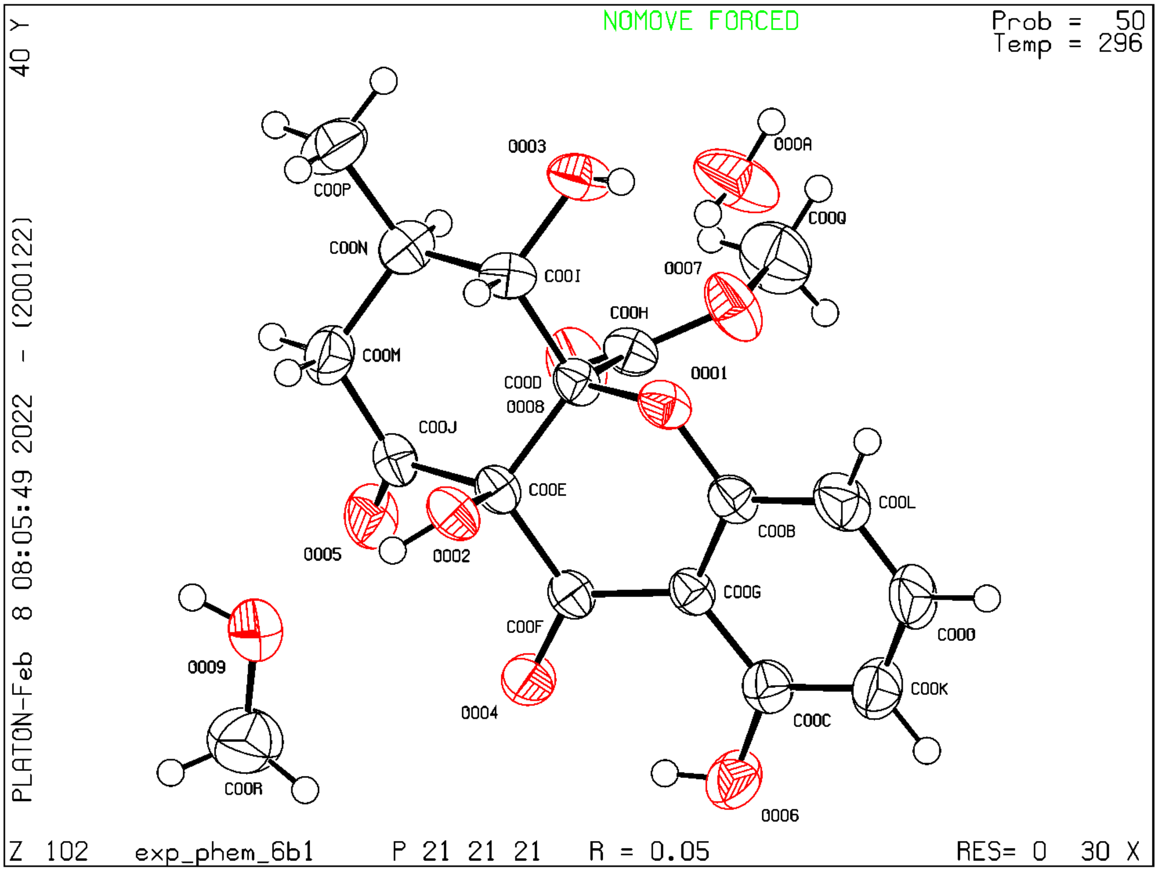

Supplement: Supplementary file 1 [file marinedrugs-20-00162-s001.zip › phem_6B-1/checkcif.pdf]
