# Supplementary material for: Amycolachromones A–F, Isolated from a Streptomycin-Resistant Strain of the Deep-Sea Marine Actinomycete Amycolatopsis sp. WP1
Source: Mar Drugs. 2022 Feb 24;20(3):162. doi: 10.3390/md20030162 (PMC8949813; doi:10.3390/md20030162)
Supplement: Supplementary file 1 [file marinedrugs-20-00162-s001.zip › phem_6B-1/phem_6B-1/phem_6B-1/struct/olex2_exp_phem_6B1/.olex/autochem_single.htm]

**exp\_phem\_6b1** (1/1)  
 (453.55294Å3, Original R1 = 0.00%)  
Solution Attempt **1**: **ShelXS/Direct Methods**  

|  |
| --- |
| **GOOD SOLUTION: Confidence in this solution 32.9** |

**48**→**56**→**56**→**56**→**CFOM**=0.071  
**Nqual**=-0.784  
**Ralpha**=0.071  
**sol\_t\_dm**=1.29  
**sol\_vss\_dm**=96  
