# Supplementary material for: Amycolachromones A–F, Isolated from a Streptomycin-Resistant Strain of the Deep-Sea Marine Actinomycete Amycolatopsis sp. WP1
Source: Mar Drugs. 2022 Feb 24;20(3):162. doi: 10.3390/md20030162 (PMC8949813; doi:10.3390/md20030162)
Supplement: Supplementary file 1 [file marinedrugs-20-00162-s001.zip › phem_6B-1/phem_6B-1/phem_6B-1/struct/olex2_exp_phem_6B1/exp_phem_6B1_cifreport.html]

checkCIF/PLATON page 2


# checkCIF (basic structural check) running

---

  
*Checking for embedded fcf data in CIF ...*
  
*No extractable fcf data in found in CIF*

# checkCIF/PLATON (basic structural check)

---


File name of structure factor file (in CIF format) on your local filesystem:

    


---

 Download CIF editor (publCIF) from the IUCr   
 Download CIF editor (enCIFer) from the CCDC   
 Test a new CIF entry 
