# Supplementary material for: Amycolachromones A–F, Isolated from a Streptomycin-Resistant Strain of the Deep-Sea Marine Actinomycete Amycolatopsis sp. WP1
Source: Mar Drugs. 2022 Feb 24;20(3):162. doi: 10.3390/md20030162 (PMC8949813; doi:10.3390/md20030162)
Supplement: Supplementary file 1 [file marinedrugs-20-00162-s001.zip › supplementary.pdf]

Article

Amycolachromones A–F, Isolated from a Streptomycin-Resistant Strain of the Deep-Sea Marine Actinomycete *Amycolatopsis* sp. WP1

Jianwei Chen [1,†], Jun Chen [2,†], Siqi Wang [1], Xiaoze Bao [1], Songwei Li [1], Bin Wei [1], Huawei Zhang [1] and Hong Wang [1,3,*]

1 College of Pharmaceutical Science & Collaborative Innovation Center of Yangtze River Delta Region Green Pharmaceuticals, Zhejiang University of Technology, Hangzhou 310014, China; cjw983617@zjut.edu.cn (J.C.); 02000179@wxc.edu.cn (J.C.); 2112007272@zjut.edu.cn (S.W.); baoxiaoze@zjut.edu.cn (X.B.); songweili93@zjut.edu.cn (S.L.); binwei@zjut.edu.cn (B.W.); hwzhang@zjut.edu.cn (H.Z.); hongw@zjut.edu.cn (H.W.);

2 College of Biotechnology and Pharmaceutical Engineering, West Anhui University, Lu'an 237499, China;

3 Key Laboratory of Marine Fishery Resources Exploitment, Utilization of Zhejiang Province, Zhejiang University of Technology, Hangzhou 310014, China;

* Correspondence: hongw@zjut.edu.cn; Tel.: +86-0571-8832-0622

† These authors contributed equally to this work.

Figure S1. ^{1}H NMR spectrum of Amycochromone A (**1**) (DMSO-d_6, 500 MHz)
Figure S2. ^{13}C NMR spectrum of Amycochromone A (**1**) (DMSO-d_6, 125 MHz)
Figure S3. COSY spectrum of Amycochromone A (**1**) (DMSO-d_6, 500 MHz)
Figure S4. HSQC spectrum of Amycochromone A (**1**) (DMSO-d_6, 500 MHz)
Figure S5. HMBC spectrum of Amycochromone A (**1**) (DMSO-d_6, 500 MHz)
Figure S6. HRESIMS spectrum of Amycochromone A (**1**)
Figure S7. ^{1}H NMR spectrum of Amycochromone B (**2**) (DMSO-d_6, 500 MHz)
Figure S8. ^{13}C NMR spectrum of Amycochromone B (**2**) (DMSO-d_6, 125 MHz)
Figure S9. COSY spectrum of Amycochromone B (**2**) (DMSO-d_6, 500 MHz)
Figure S10. HSQC spectrum of Amycochromone B (**2**) (DMSO-d_6, 500 MHz)
Figure S11. HMBC spectrum of Amycochromone B (**2**) (DMSO-d_6, 500 MHz)
Figure S12. HRESIMS spectrum of Amycochromone B (**2**)
Figure S13. ^{1}H NMR spectrum of Amycochromone C (**3**) (DMSO-d_6, 500 MHz)
Figure S14. ^{13}C NMR spectrum of Amycochromone C (**3**) (DMSO-d_6, 125 MHz)
Figure S15. COSY spectrum of Amycochromone C (**3**) (DMSO-d_6, 500 MHz)
Figure S16. HSQC spectrum of Amycochromone C (**3**) (DMSO-d_6, 500 MHz)
Figure S17. HMBC spectrum of Amycochromone C (**3**) (DMSO-d_6, 500 MHz)
Figure S18. HRESIMS spectrum of Amycochromone C (**3**)
Figure S19. ^{1}H NMR spectrum of Amycochromone D (**4**) (DMSO-d_6, 500 MHz)
Figure S20. ^{13}C NMR spectrum of Amycochromone D (**4**) (DMSO-d_6, 125 MHz)
Figure S21. HSQC spectrum of Amycochromone D (**4**) (DMSO-d_6, 500 MHz)
Figure S22. HMBC spectrum of Amycochromone D (**4**) (DMSO-d_6, 500 MHz)
Figure S23. HRESIMS spectrum of Amycochromone D (**4**)
Figure S24. ^{1}H NMR spectrum of Amycochromone E (**5**) (DMSO-d_6, 500 MHz)
Figure S25. ^{13}C NMR spectrum of Amycochromone E (**5**) (DMSO-d_6, 125 MHz)
Figure S26. COSY spectrum of Amycochromone E (**5**) (DMSO-d_6, 500 MHz)
Figure S27. HSQC spectrum of Amycochromone E (**5**) (DMSO-d_6, 500 MHz)
Figure S28. HMBC spectrum of Amycochromone E (**5**) (DMSO-d_6, 500 MHz)
Figure S29. HRESIMS spectrum of Amycochromone E (**5**)
Figure S30. ^{1}H NMR spectrum of Amycochromone F (**6**) (DMSO-d_6, 500 MHz)
Figure S31. ^{13}C NMR spectrum of Amycochromone F (**6**) (DMSO-d_6, 125 MHz)
Figure S32. COSY spectrum of Amycochromone F (**6**) (DMSO-d_6, 500 MHz)
Figure S33. HSQC spectrum of Amycochromone F (**6**) (DMSO-d_6, 500 MHz)

Figure S34. HMBC spectrum of Amycochromone F (**6**) (DMSO-d_6, 500 MHz)
Figure S35. HRESIMS spectrum of Amycochromone F (**6**)
Table S1. Crystallographic data for Amycochromone F (**6**)

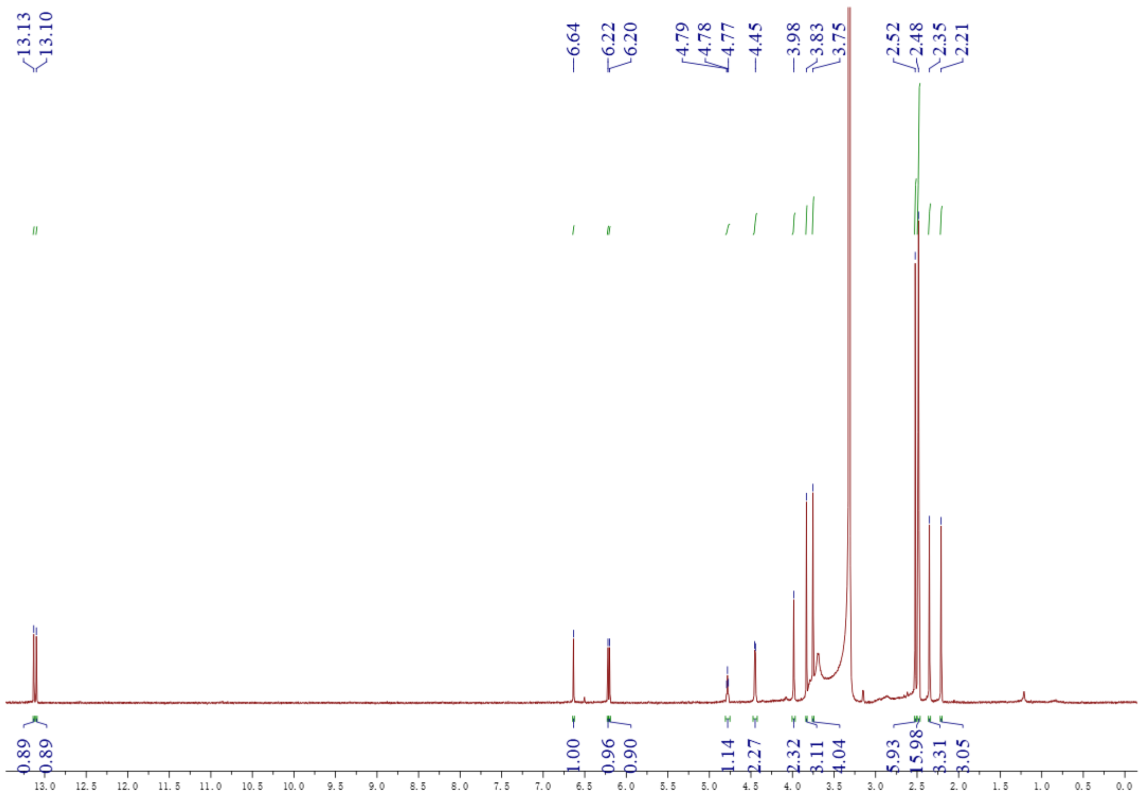

Figure S1. ^{1}H NMR spectrum of Amycochromone A (**1**) (DMSO-d_6, 500 MHz)

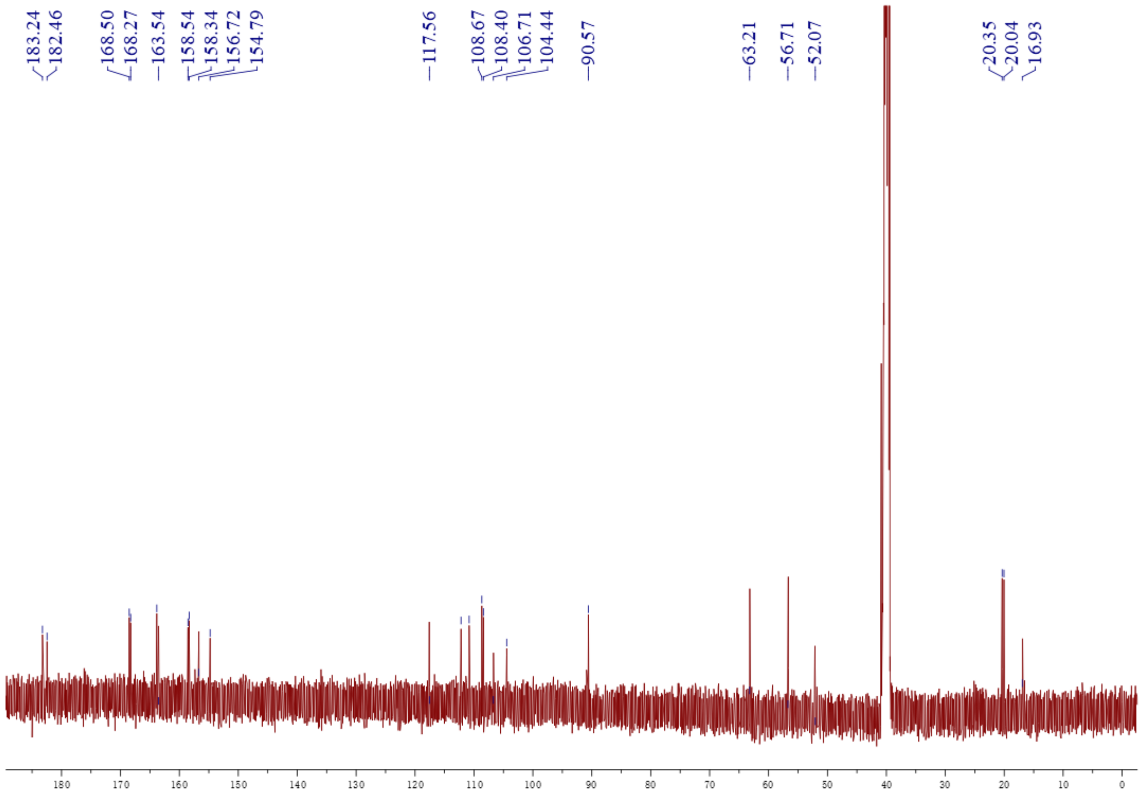

Figure S2. ^{13}C NMR spectrum of Amycochromone A (**1**) (DMSO-d_6, 125 MHz)

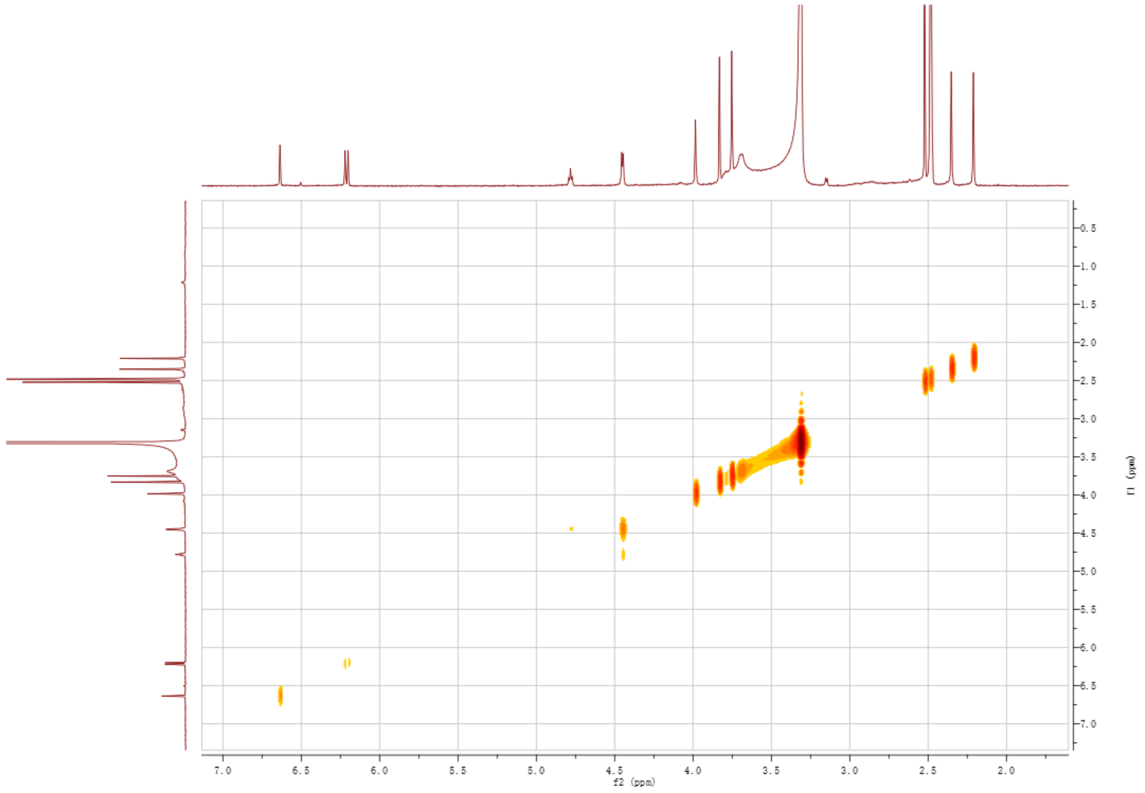

Figure S3. COSY spectrum of Amycochromone A (**1**) (DMSO-d_6, 500 MHz)

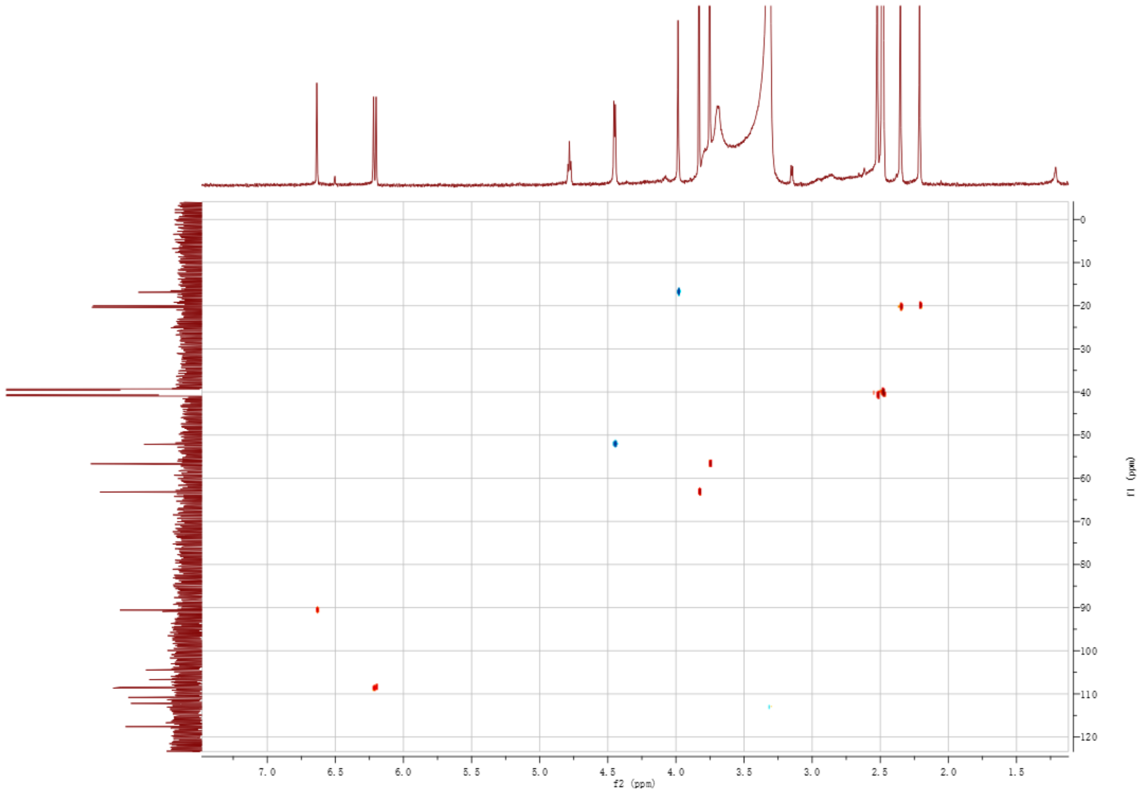

Figure S4. HSQC spectrum of Amycochromone A (**1**) (DMSO-d_6, 500 MHz)

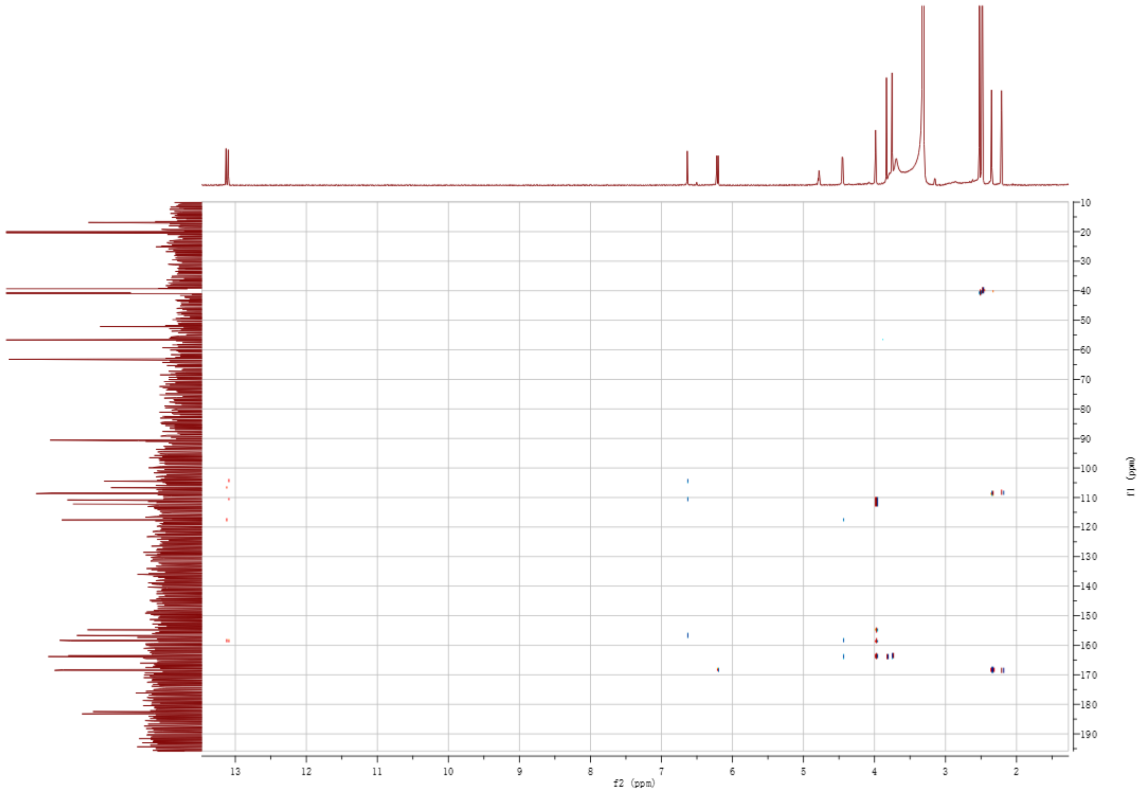

Figure S5. HMBC spectrum of Amycochromone A (**1**) (DMSO-d_6, 500 MHz)

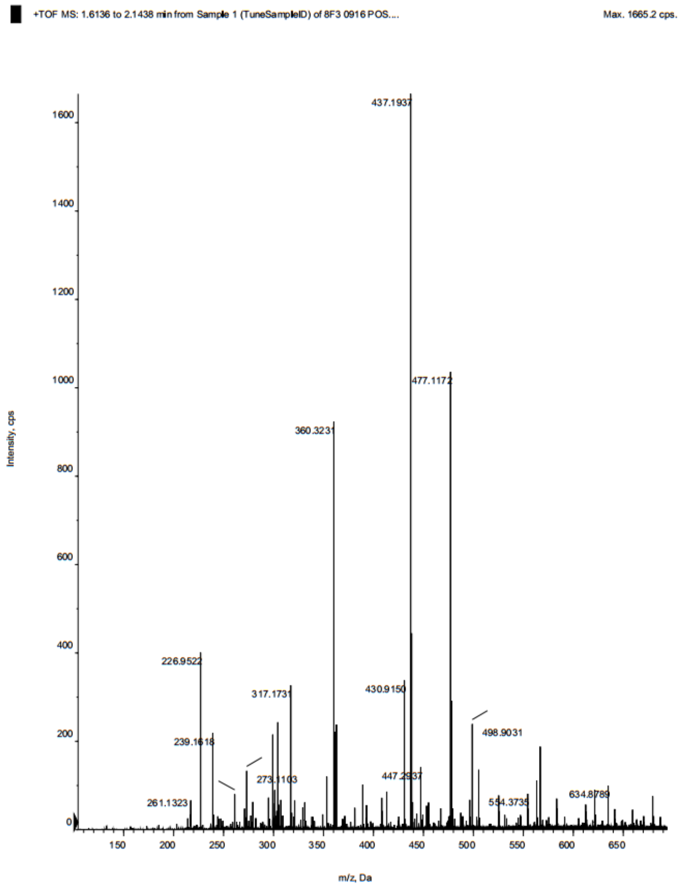

Figure S6. HRESIMS spectrum of Amycochromone A (**1**)

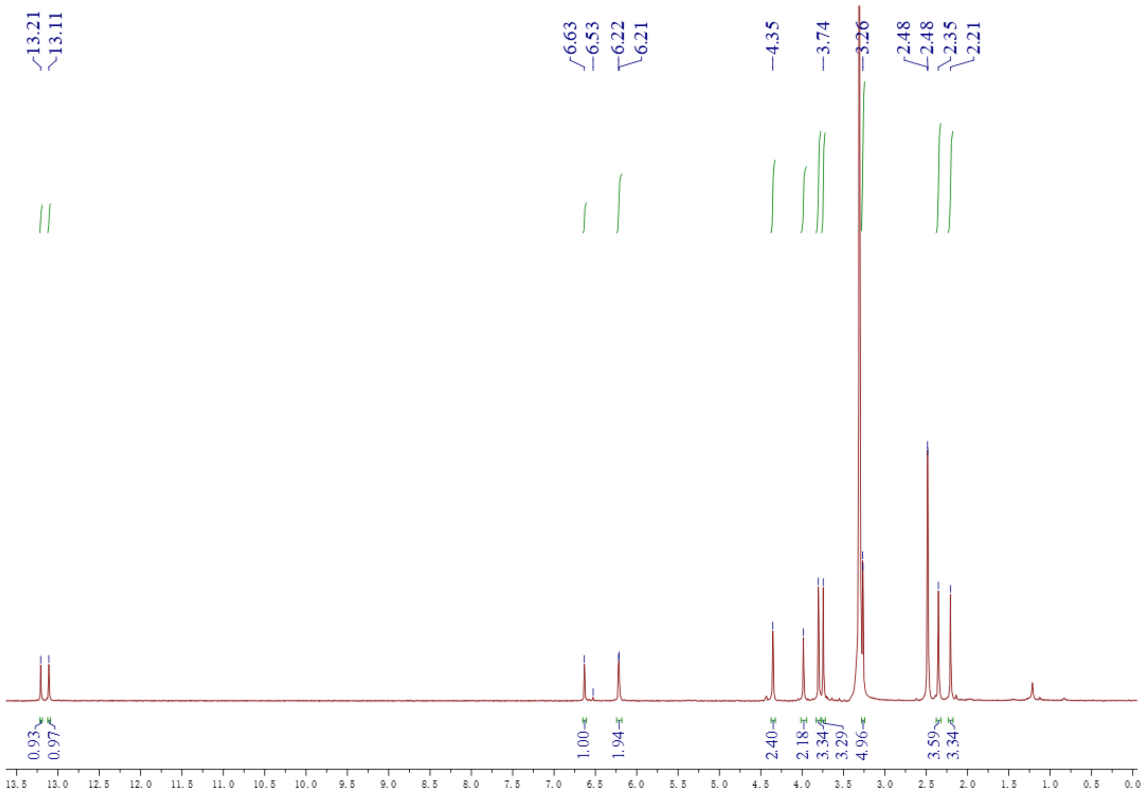

Figure S7. ^{1}H NMR spectrum of Amycochromone B (**2**) (DMSO-d_6, 500 MHz)

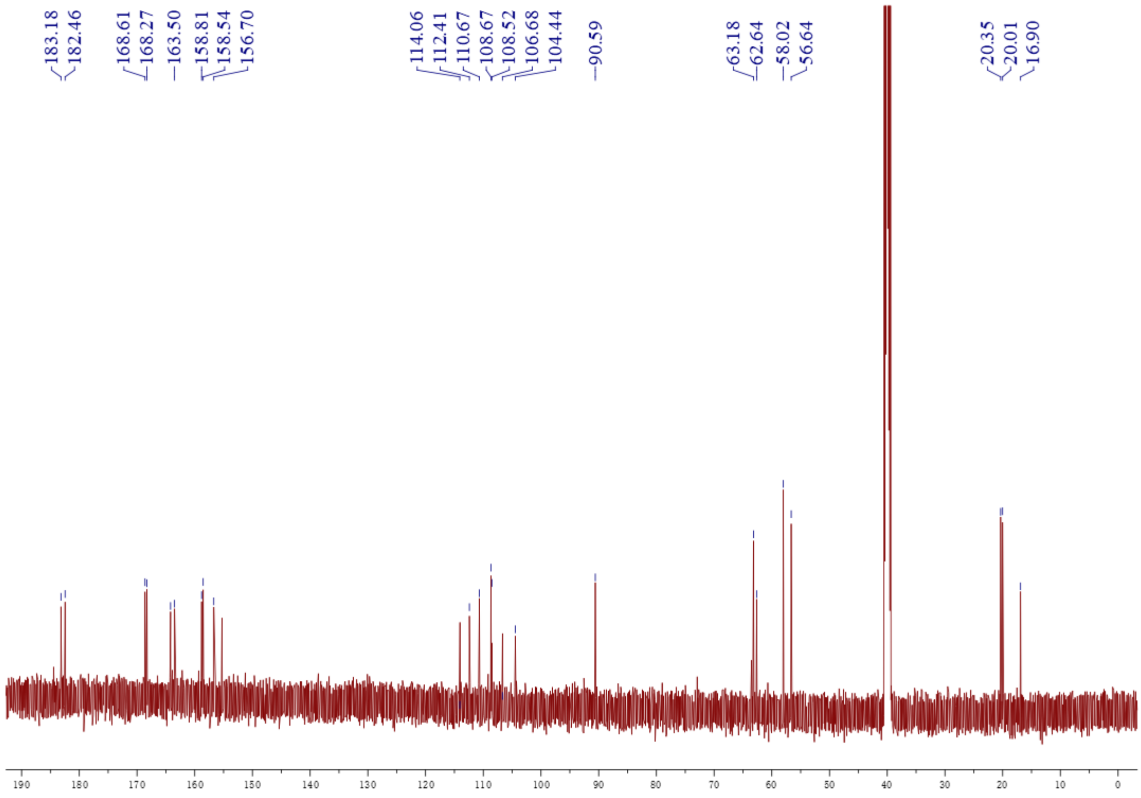

Figure S8. ^{13}C NMR spectrum of Amycochromone B (**2**) (DMSO-d_6, 125 MHz)

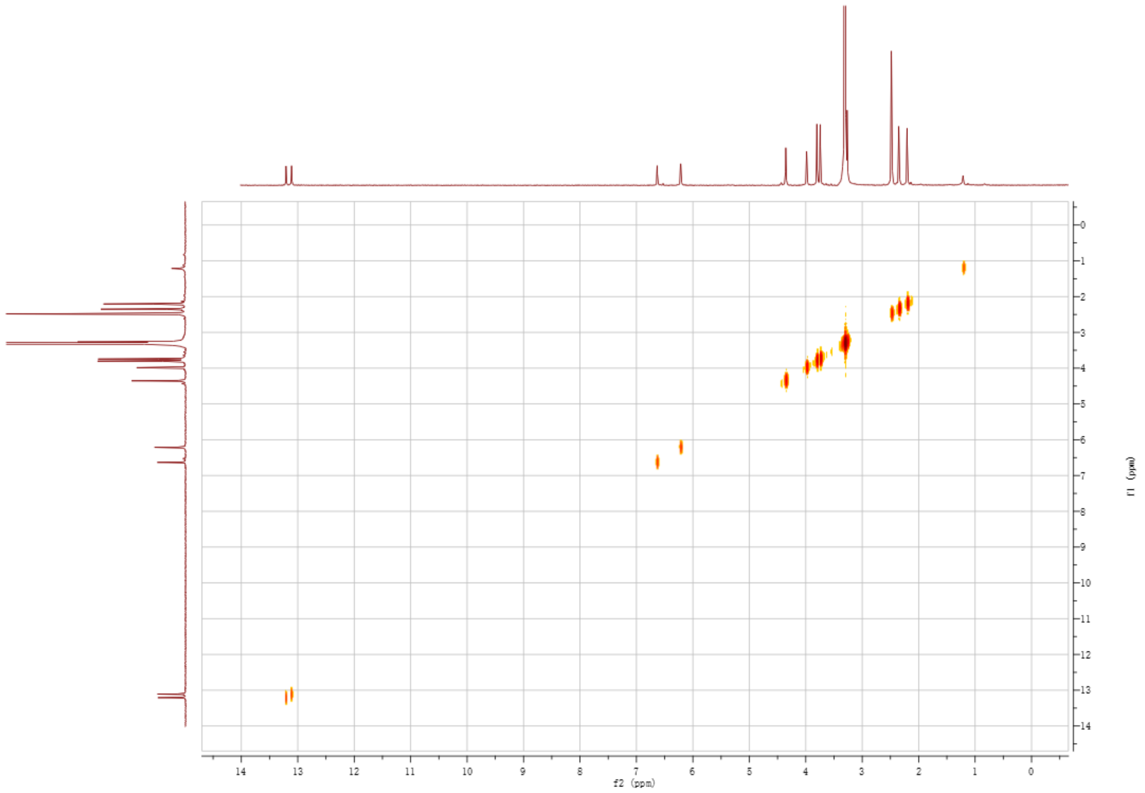

Figure S9. COSY spectrum of Amycochromone B (**2**) (DMSO-d_6, 500 MHz)

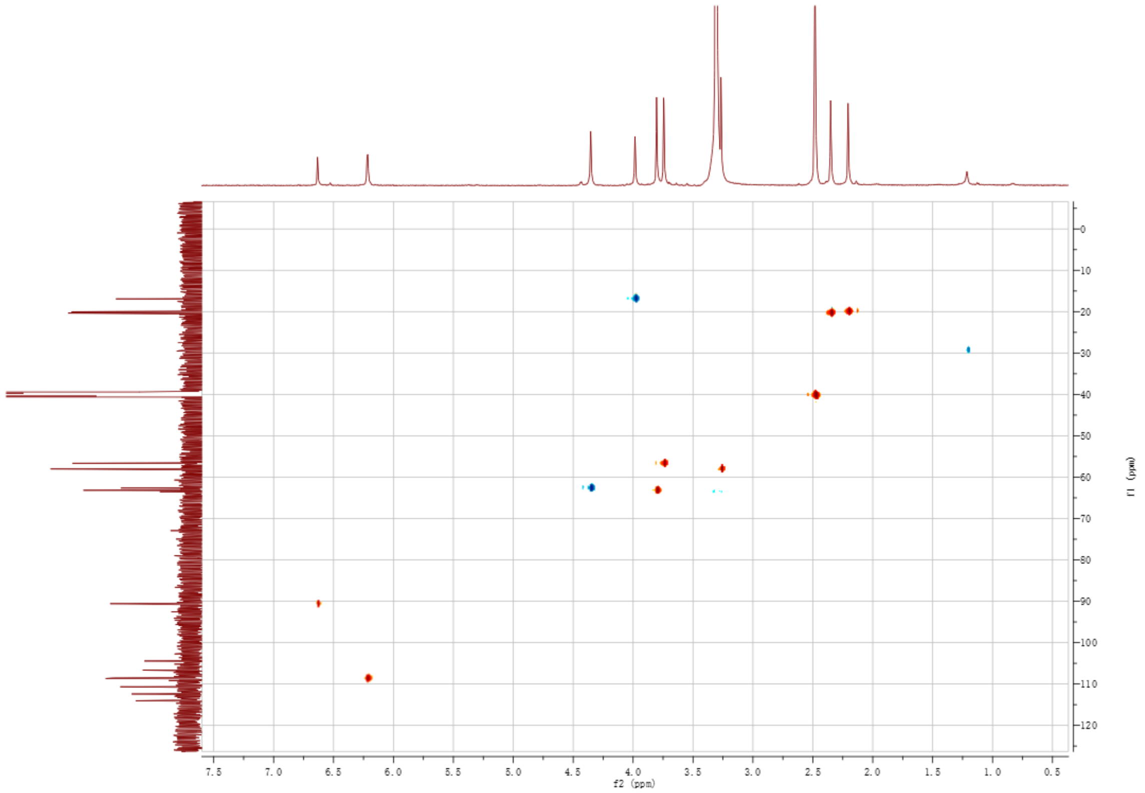

Figure S10. HSQC spectrum of Amycochromone B (**2**) (DMSO-*d*6, 500 MHz)

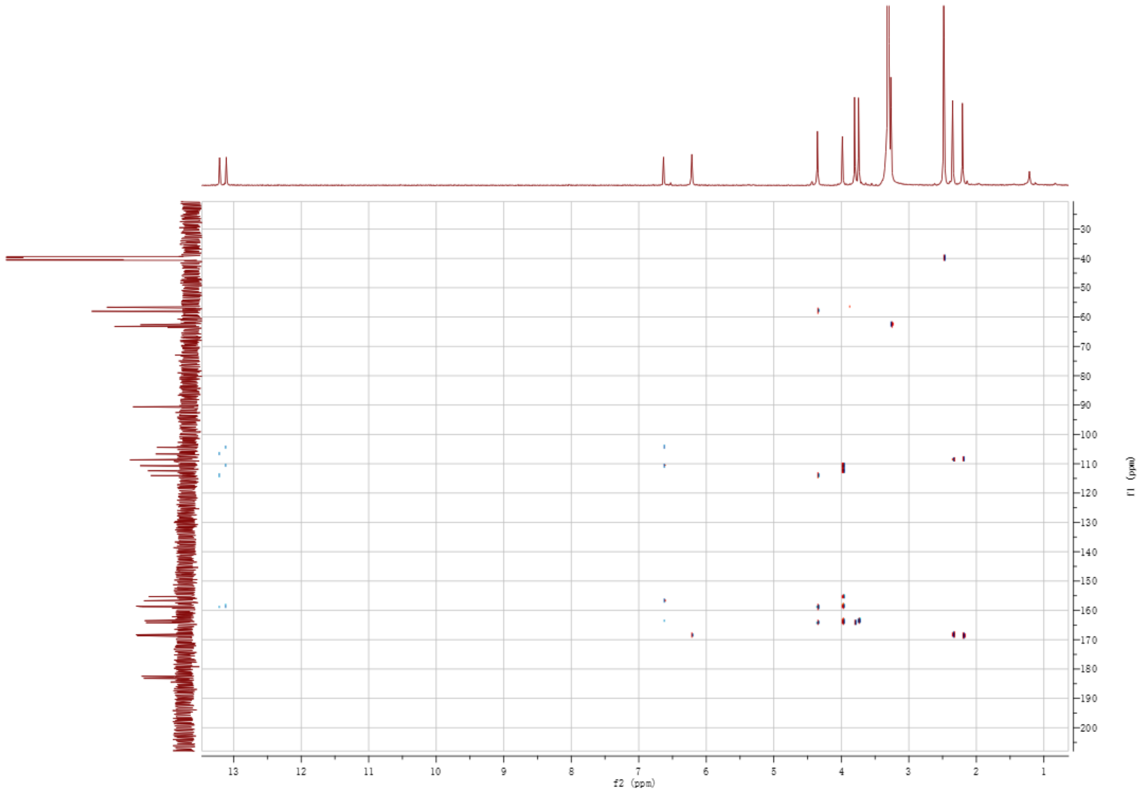

Figure S11. HMBC spectrum of Amycochromone B (**2**) (DMSO-*d*6, 500 MHz)

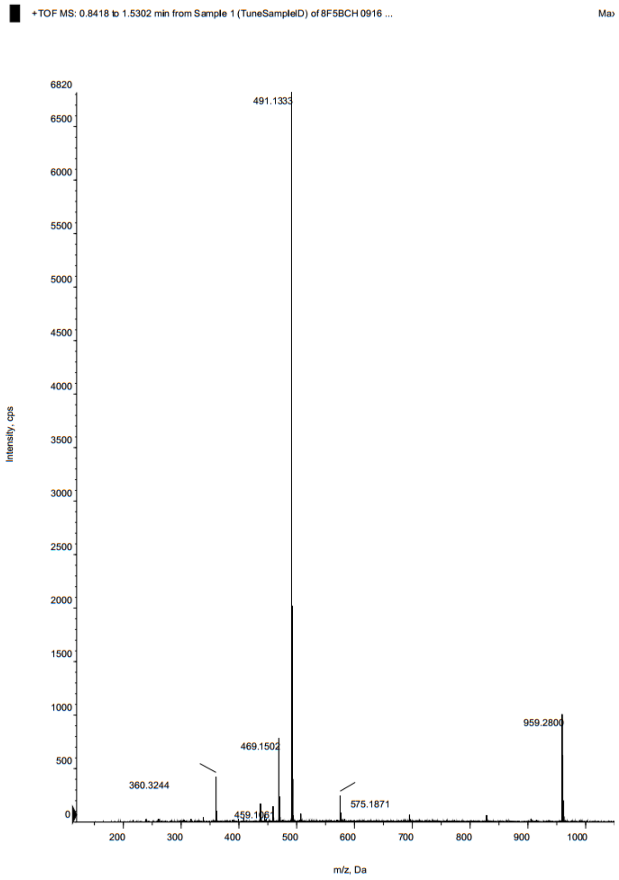

Figure S12. HRESIMS spectrum of Amycochromone B (**2**)

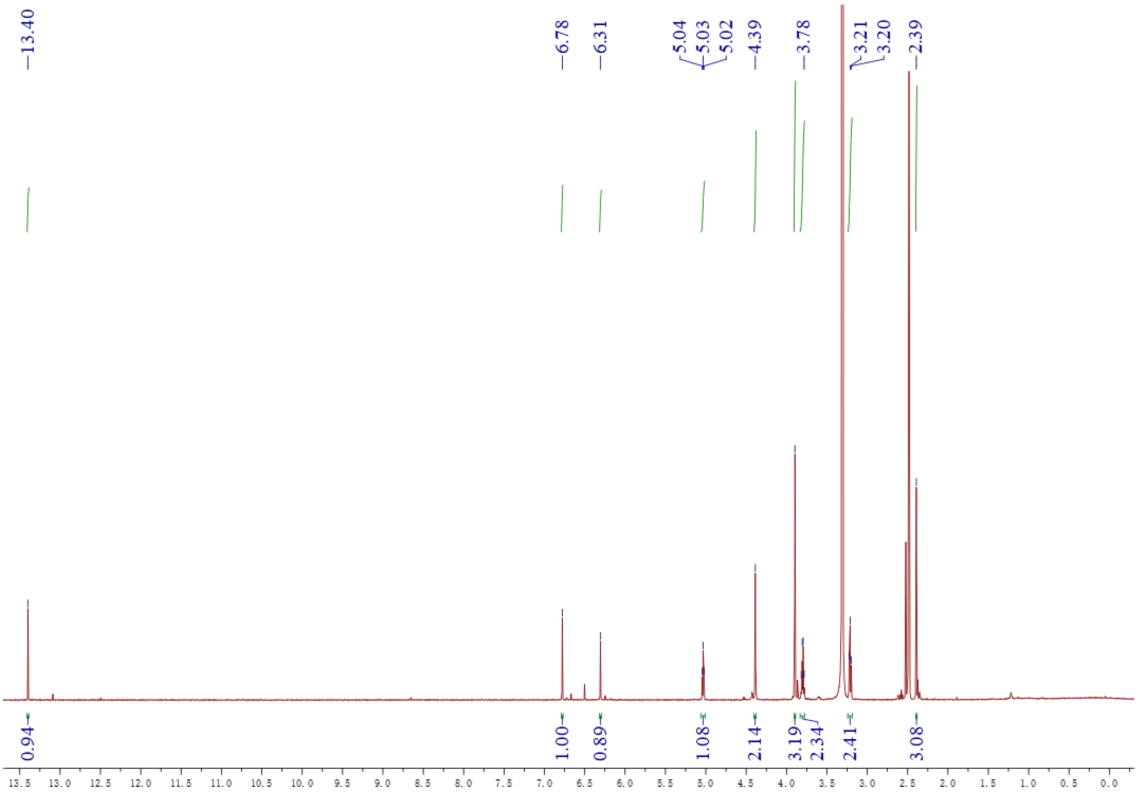

Figure S13. ^1H NMR spectrum of Amycochromone C (**3**) (DMSO-d_6, 500 MHz)

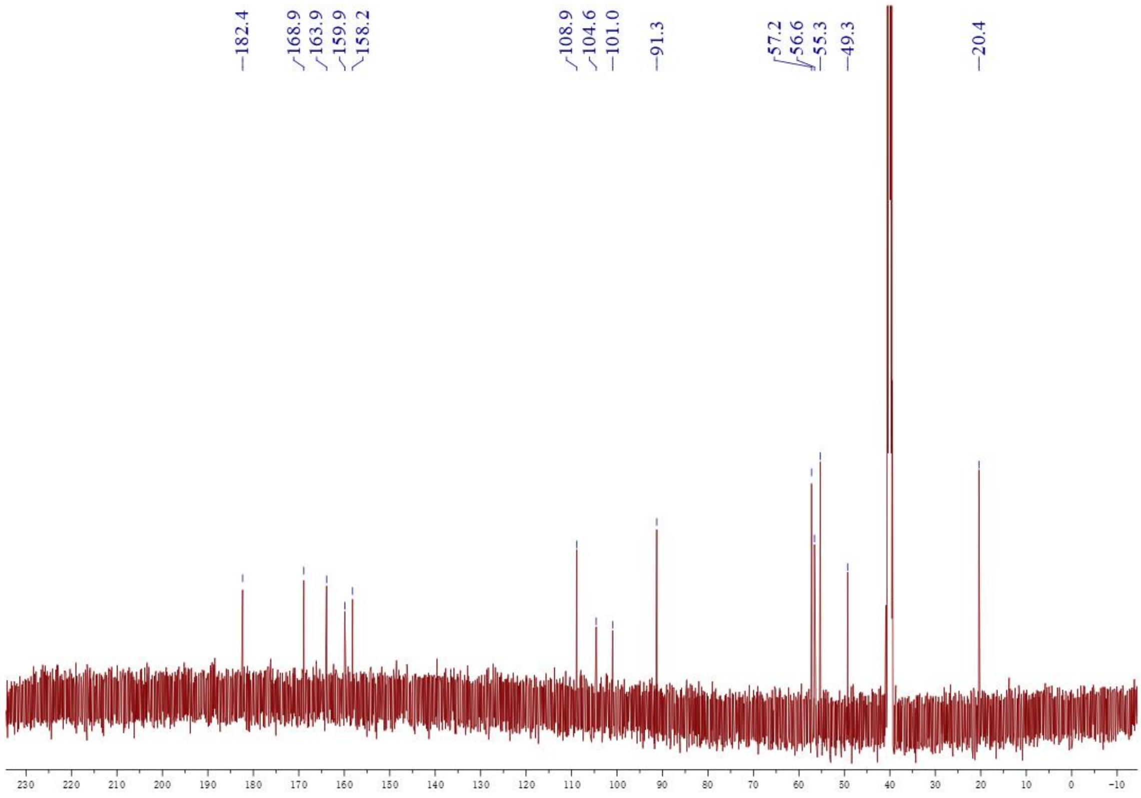

Figure S14. ^{13}C NMR spectrum of Amycochromone C (**3**) (DMSO-d_6, 125 MHz)

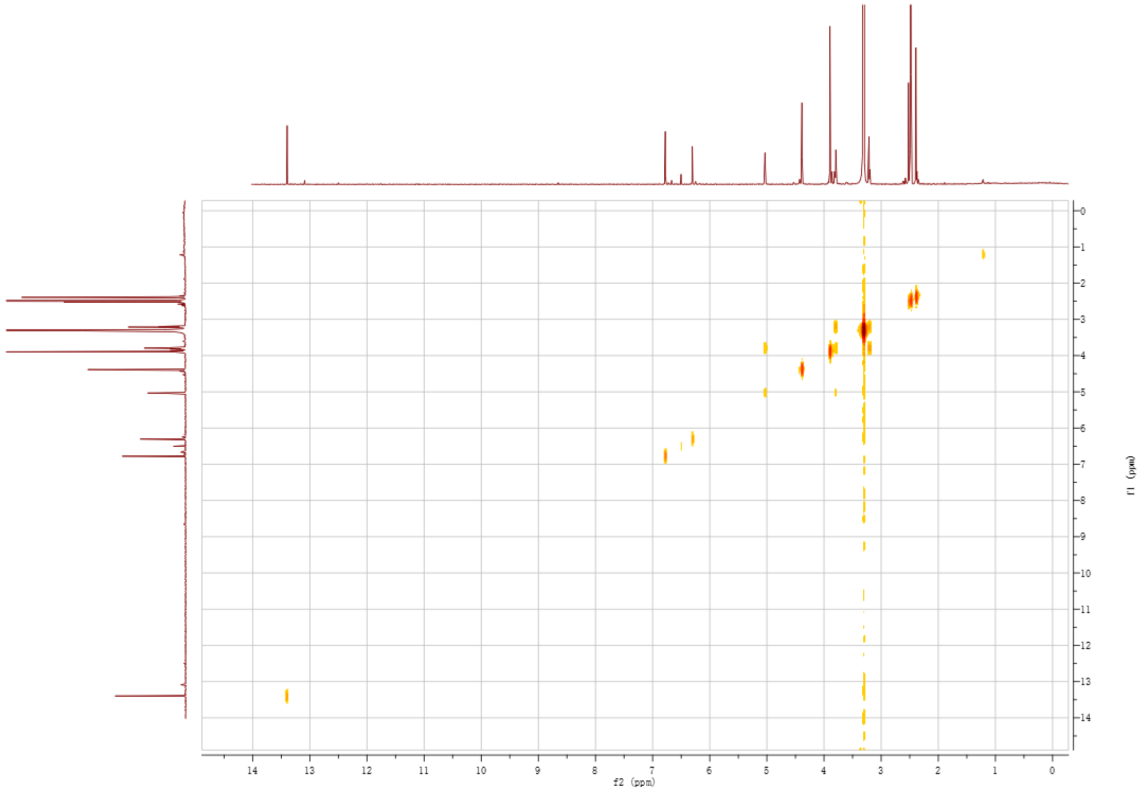

Figure S15. COSY spectrum of Amycochromone C (**3**) (DMSO-d_6, 500 MHz)

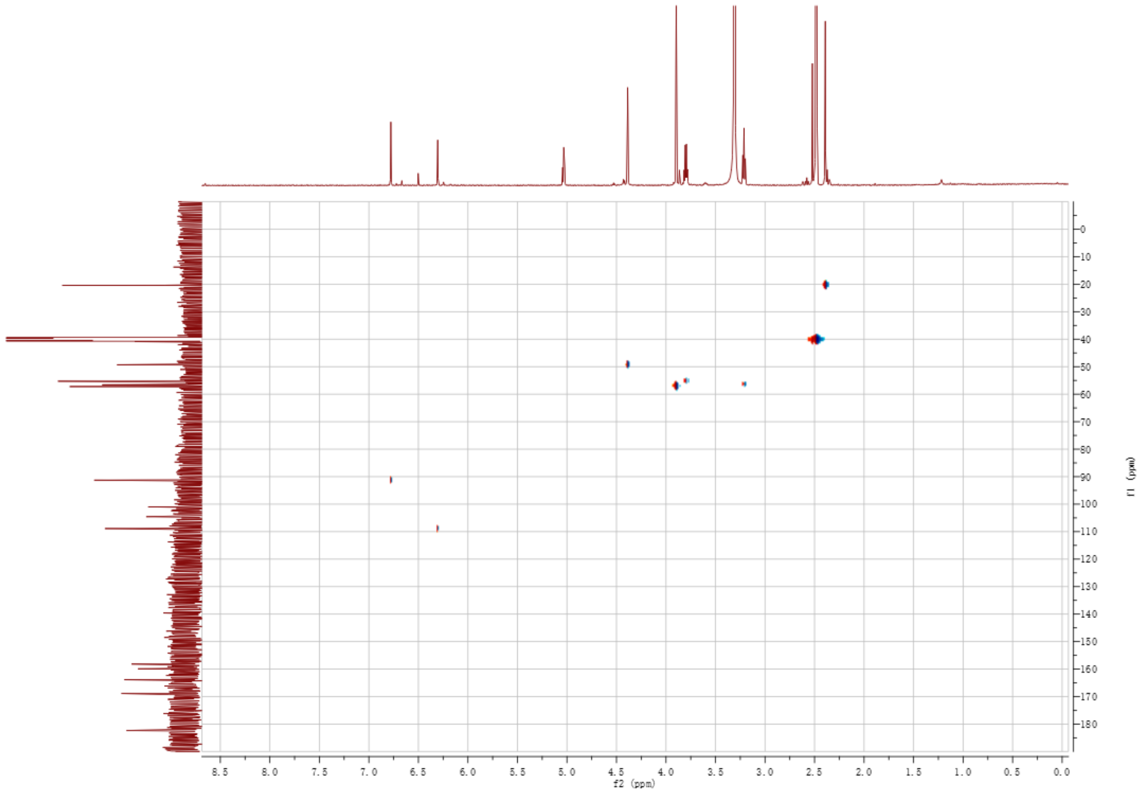

Figure S16. HSQC spectrum of Amycochromone C (**3**) (DMSO-d_6, 500 MHz)

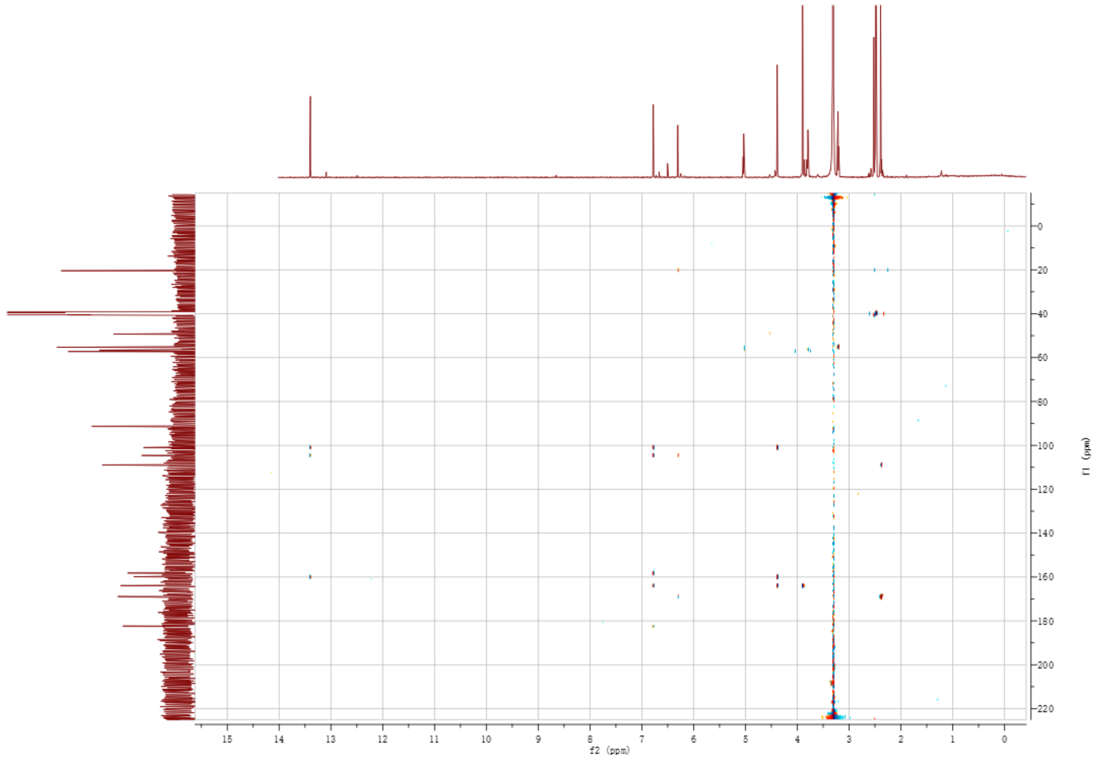

Figure S17. HMBC spectrum of Amycochromone C (**3**) (DMSO-d_6, 500 MHz)

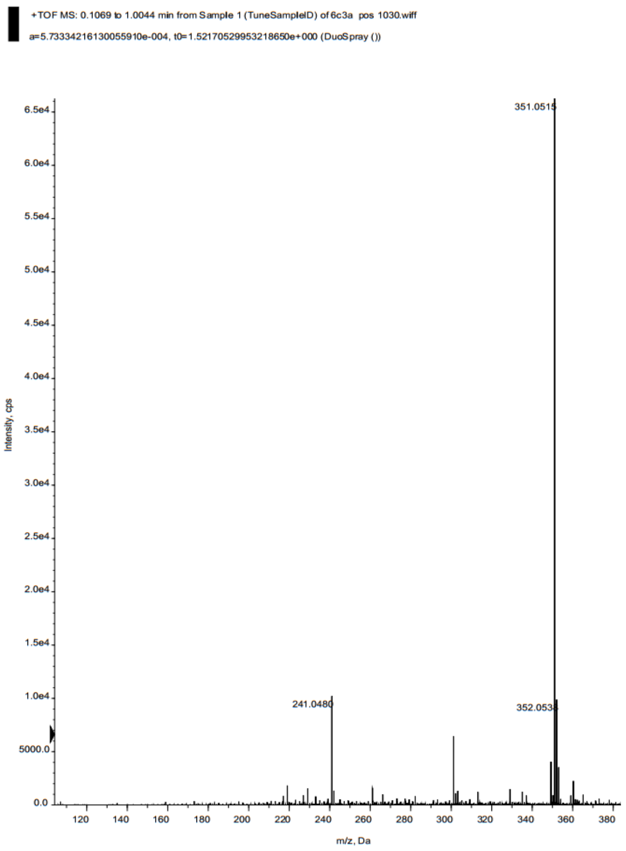

Figure S18. HRESIMS spectrum of Amycochromone C (**3**)

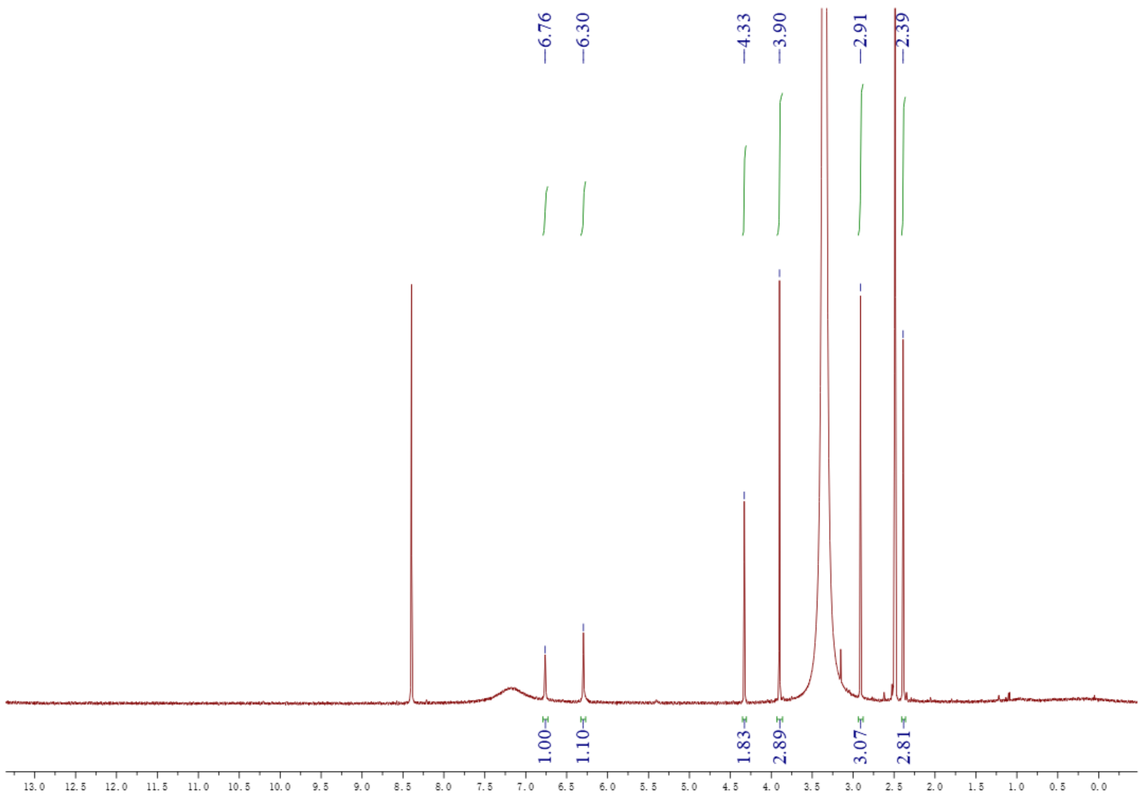

Figure S19. ^{1}H NMR spectrum of Amycochromone D (**4**) (DMSO-d_6, 500 MHz)

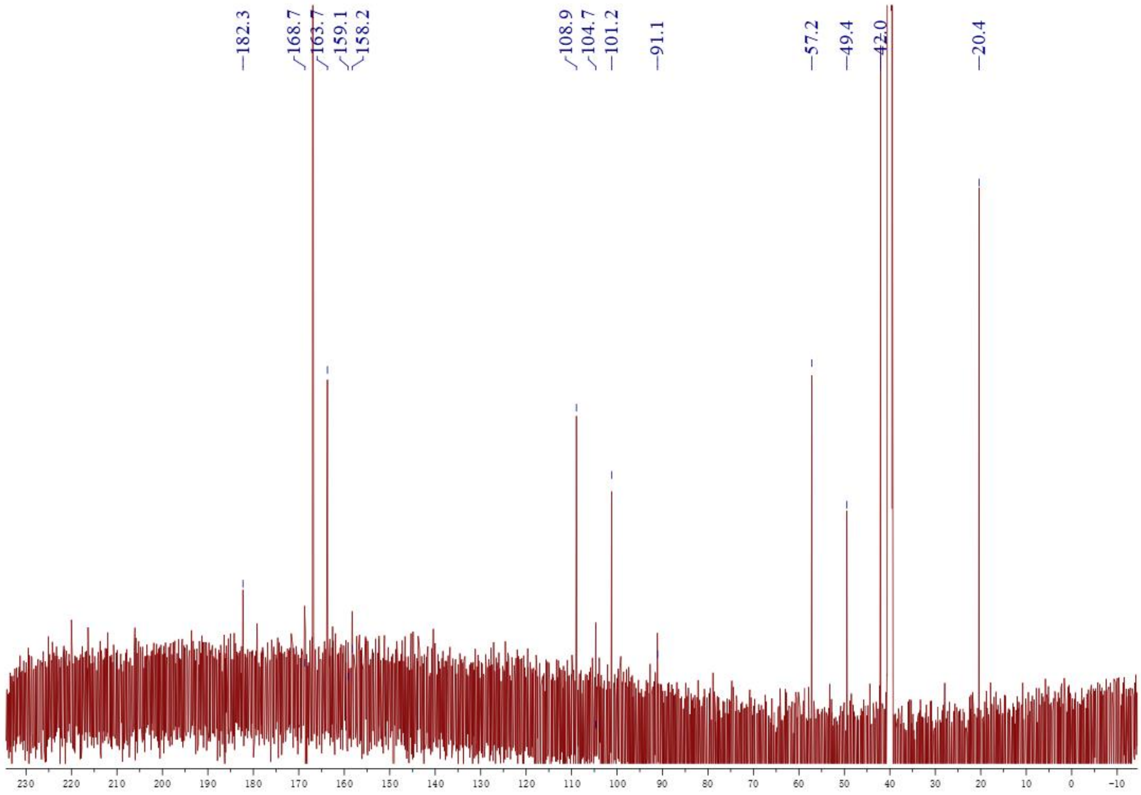

Figure S20. ^{13}C NMR spectrum of Amycochromone D (**4**) (DMSO-d_6, 125 MHz)

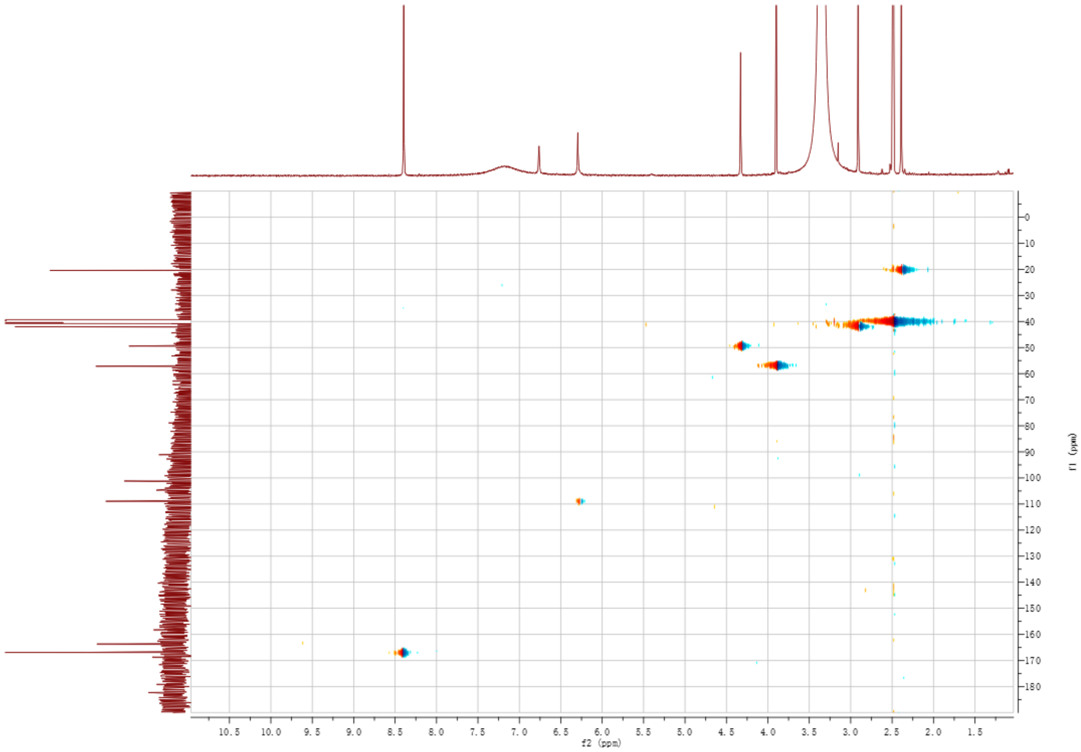

Figure S21. HSQC spectrum of Amycochromone D (**4**) (DMSO-d_6, 500 MHz)

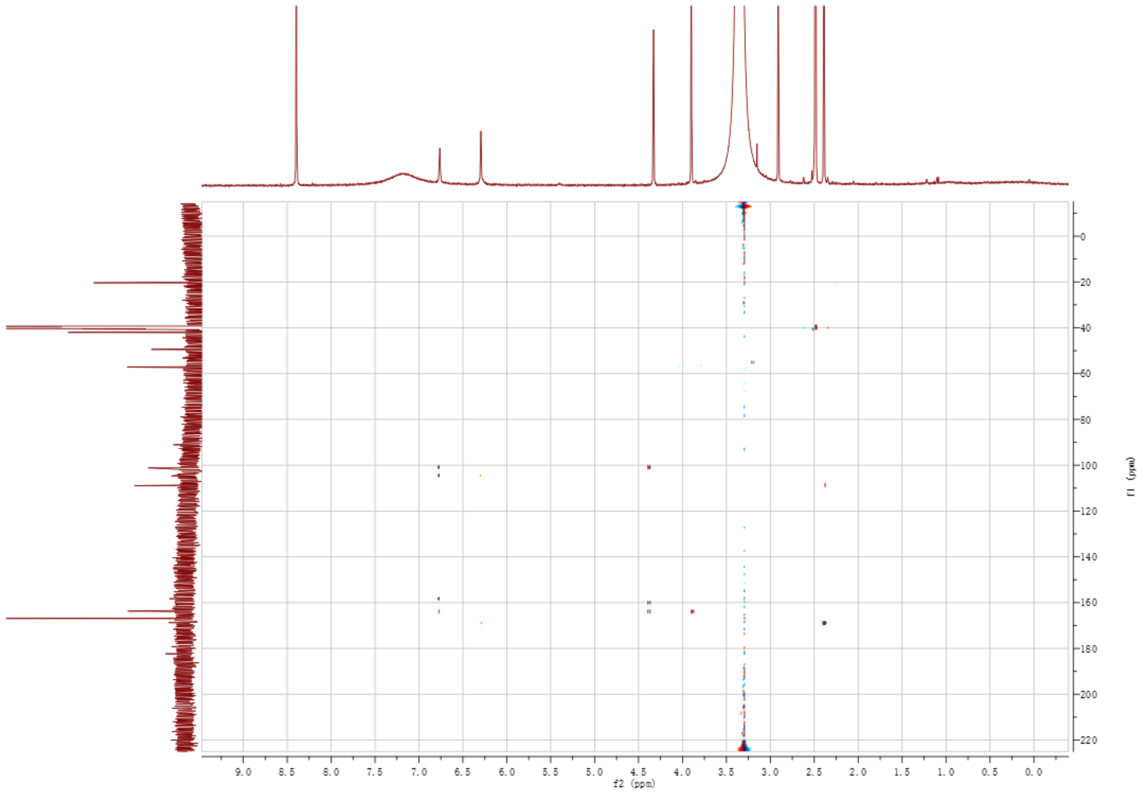

Figure S22. HMBC spectrum of Amycochromone D (**4**) (DMSO-d_6, 500 MHz)

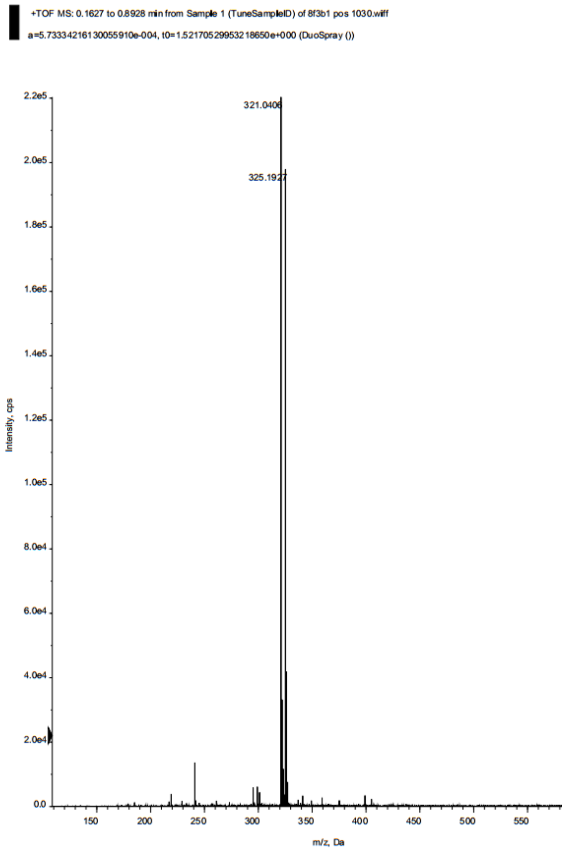

Figure S23. HRESIMS spectrum of Amycochromone D (**4**)

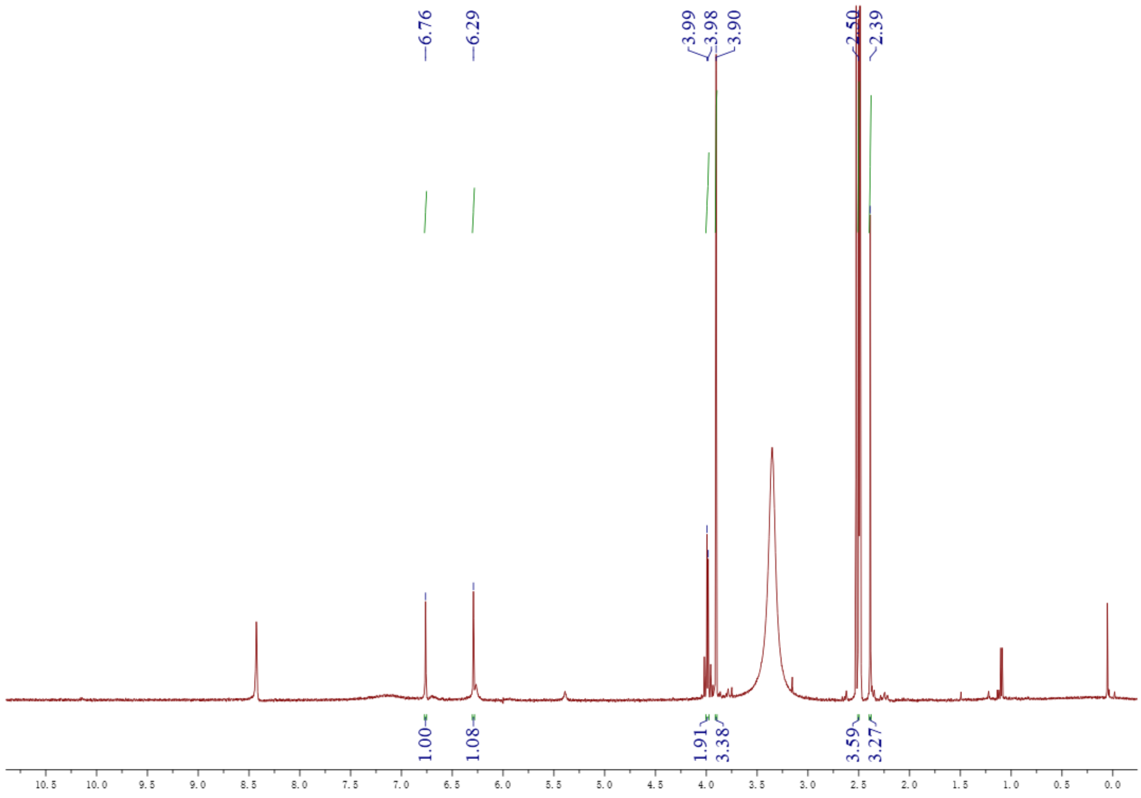

Figure S24. ¹H NMR spectrum of Amycochromone E (**5**) (DMSO-d_6, 500 MHz)

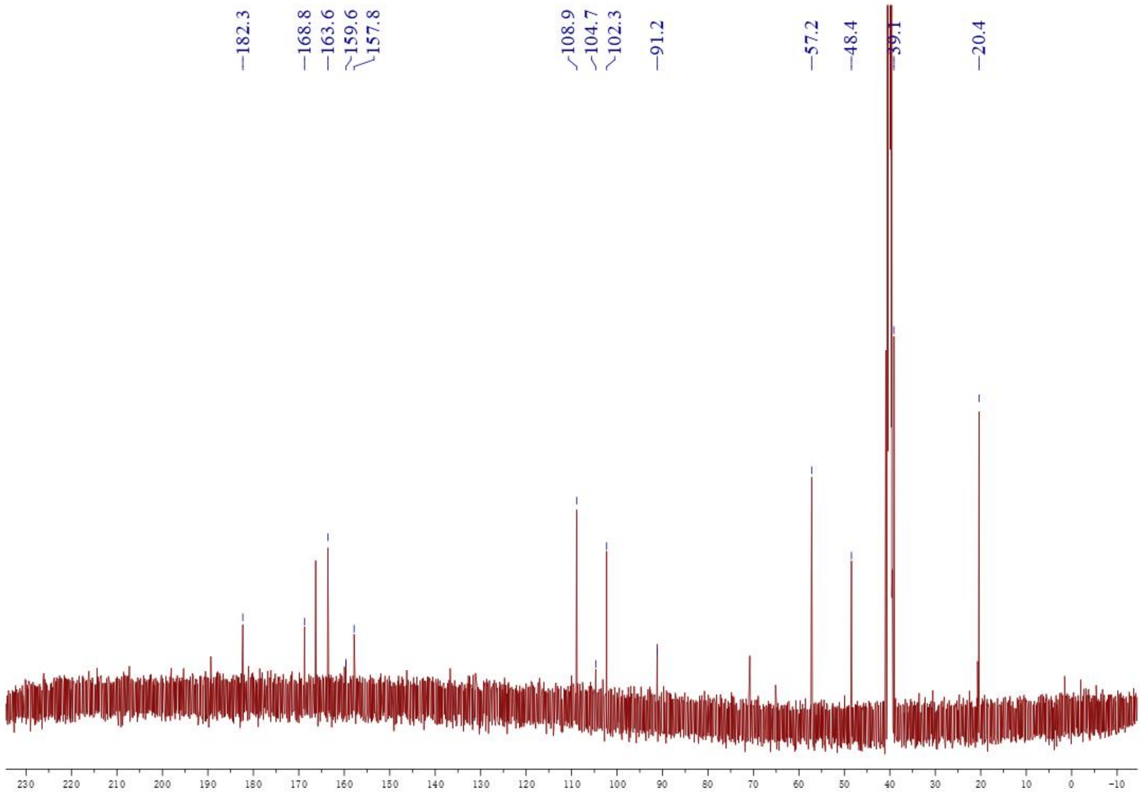

Figure S25. ¹³C NMR spectrum of Amycochromone E (**5**) (DMSO-d_6, 125 MHz)

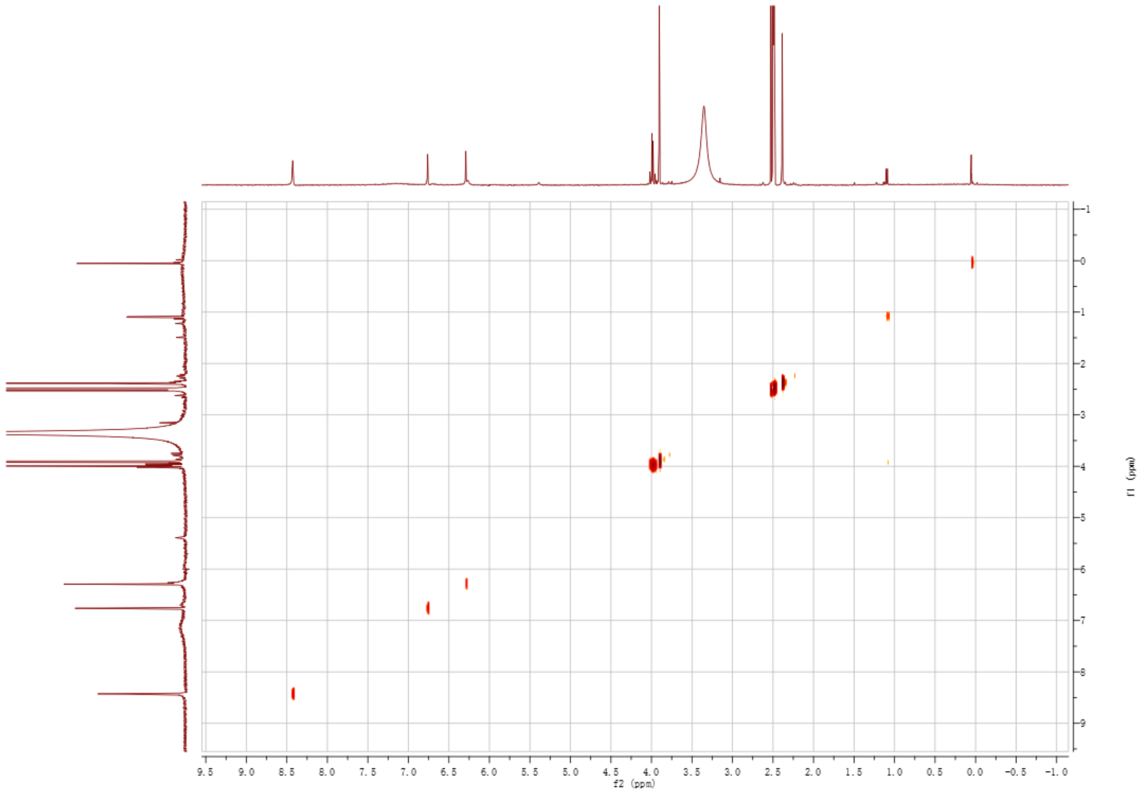

Figure S26. COSY spectrum of Amycochromone E (**5**) (DMSO-d_6, 500 MHz)

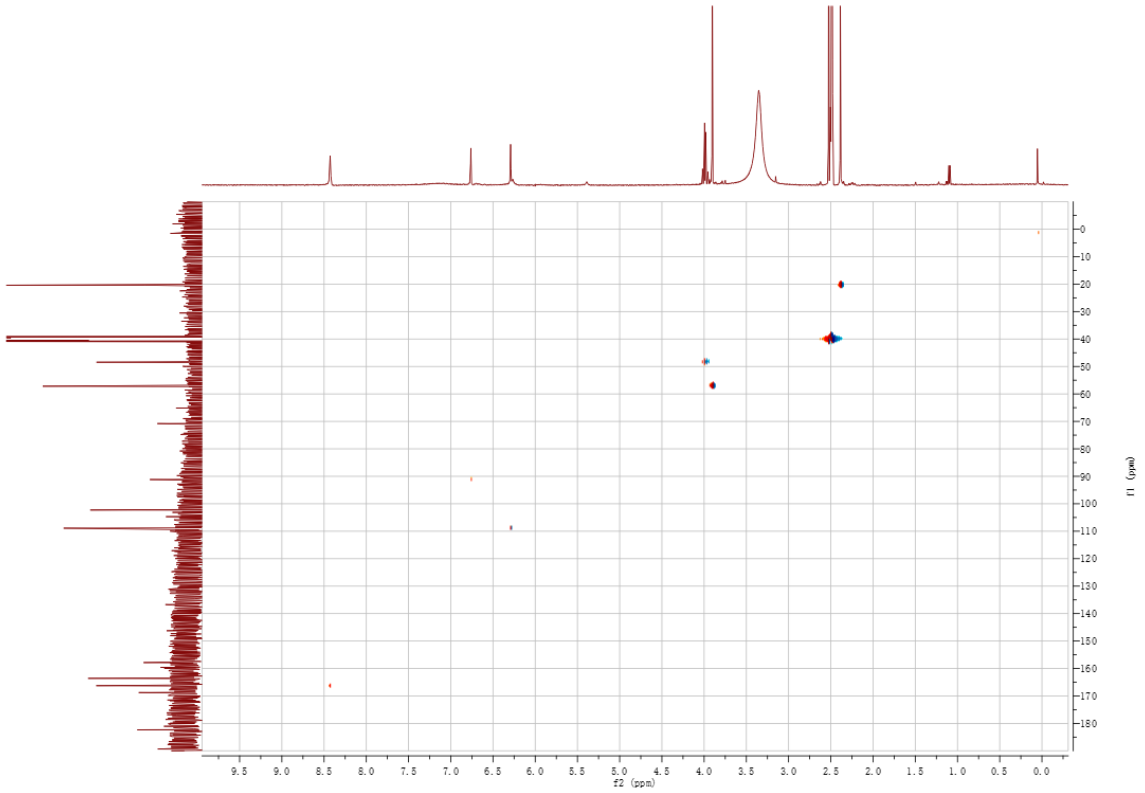

Figure S27. HSQC spectrum of Amycochromone E (**5**) (DMSO-d_6, 500 MHz)

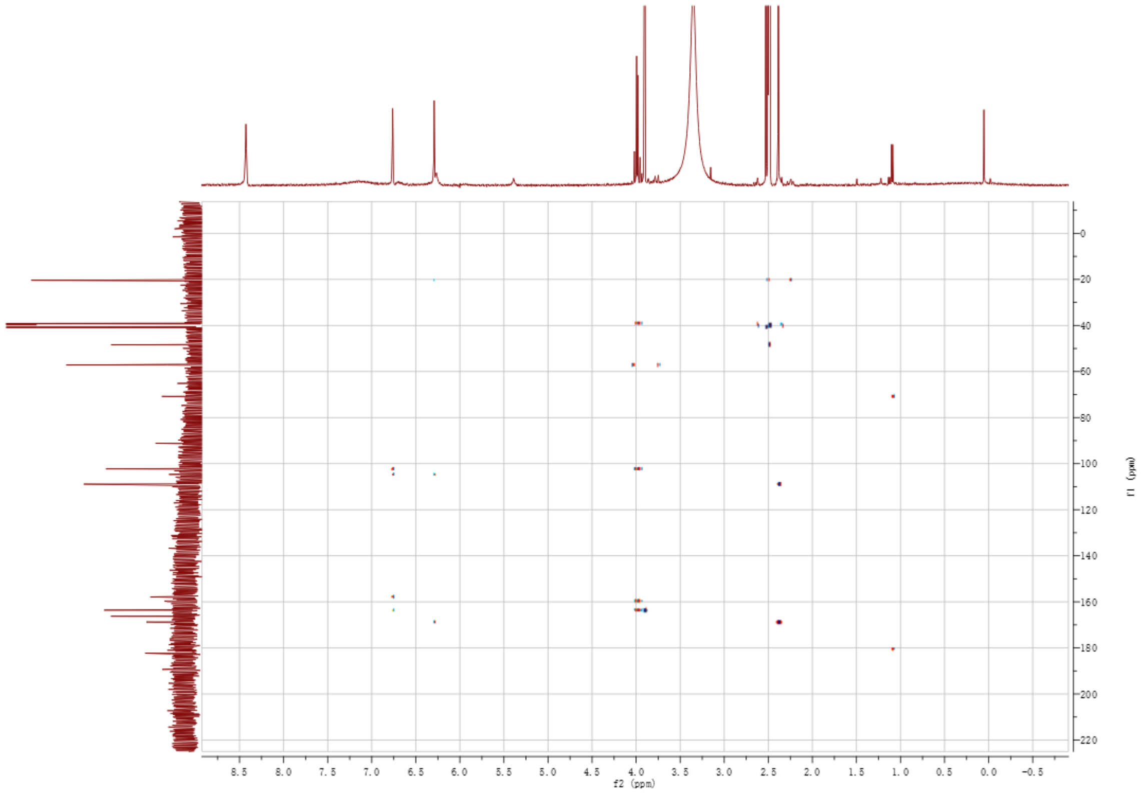

Figure S28. HMBC spectrum of Amycochromone E (**5**) (DMSO-d_6, 500 MHz)

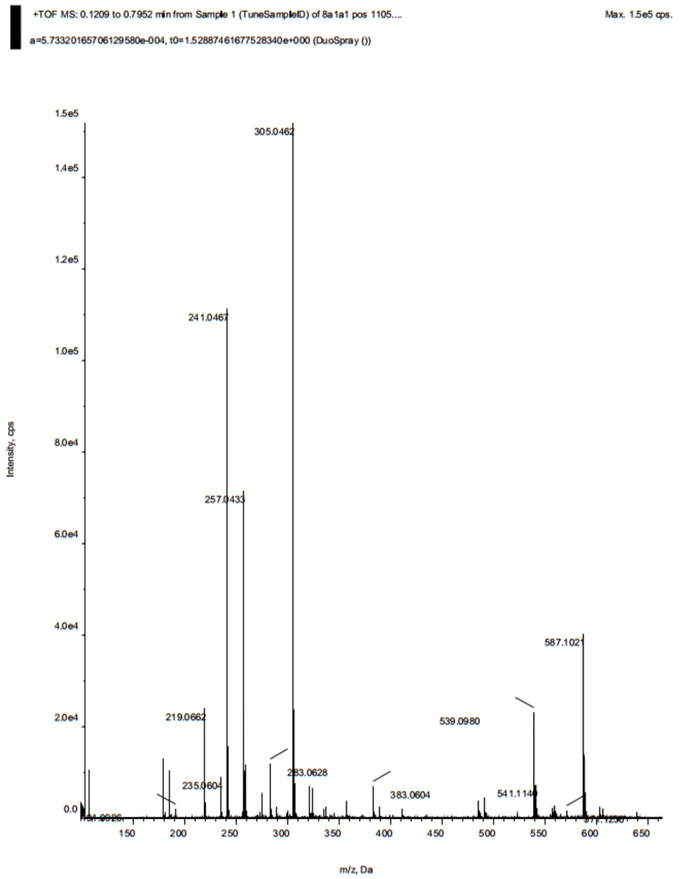

Figure S29. HRESIMS spectrum of Amycochromone E (**5**)

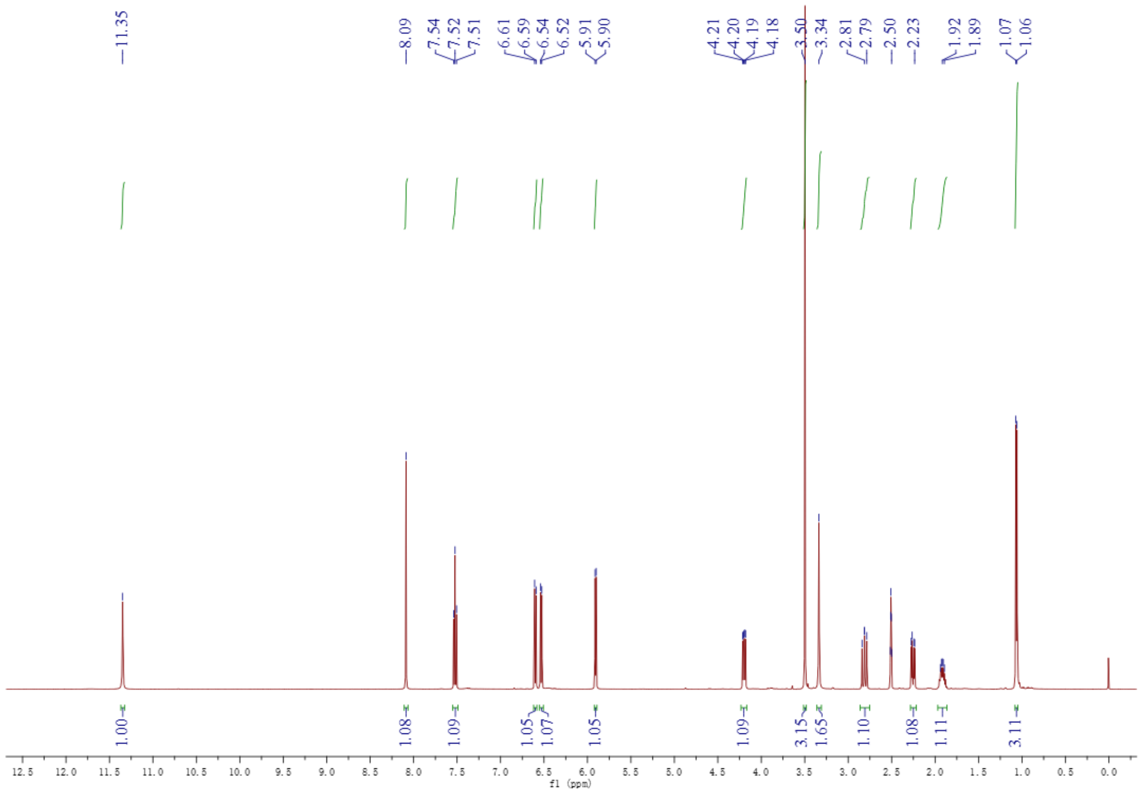

Figure S30. ^{1}H NMR spectrum of Amycochromone F (**6**) (DMSO-d_6, 500 MHz)

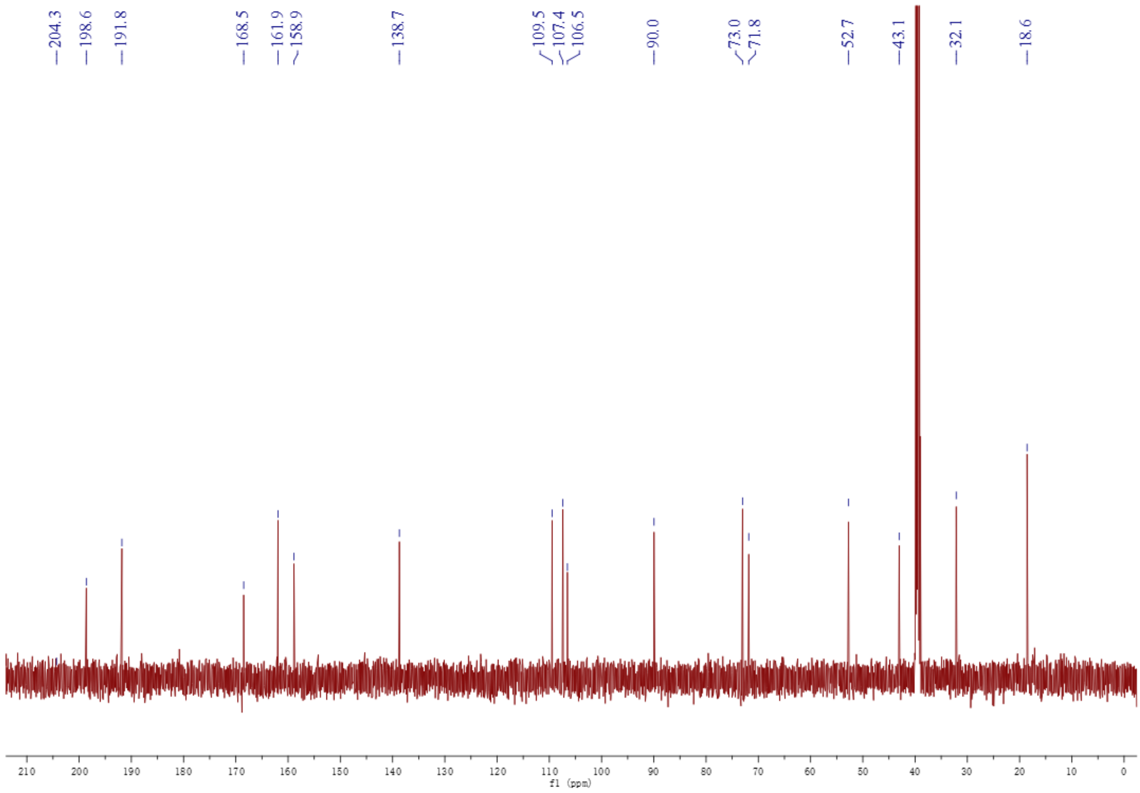

Figure S31. ^{13}C NMR spectrum of Amycochromone F (**6**) (DMSO-d_6, 125 MHz)

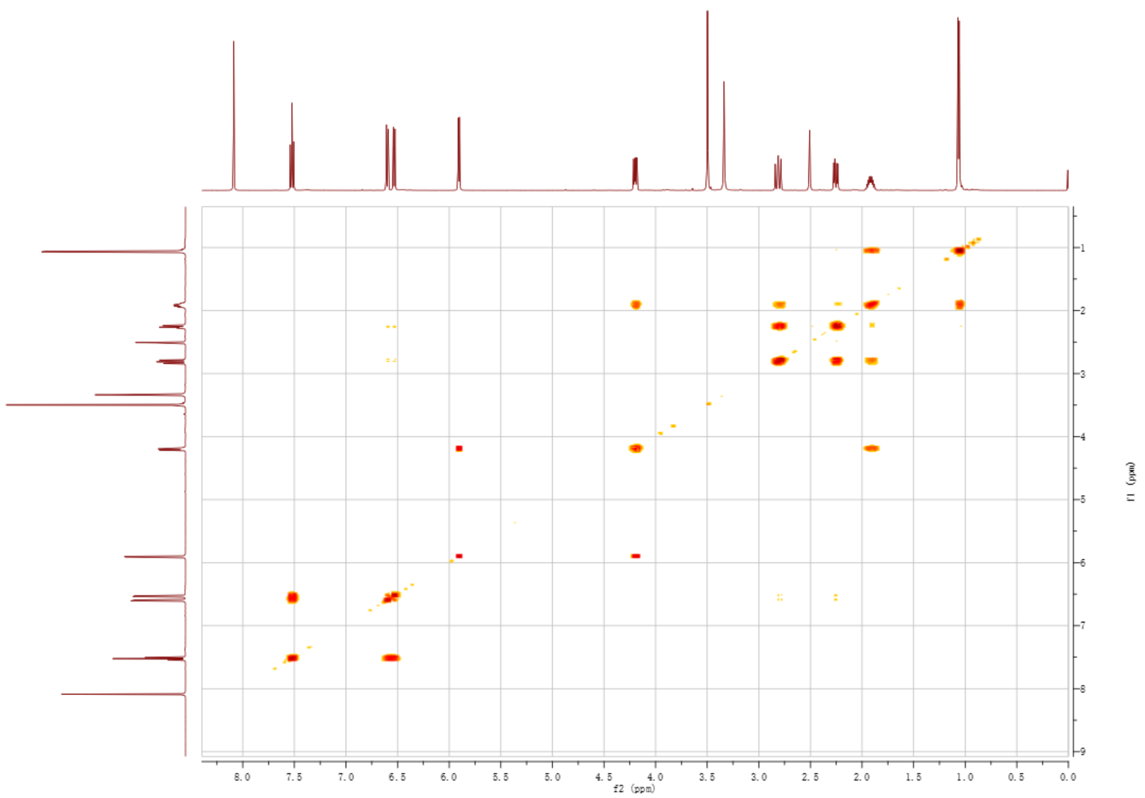

Figure S32. COSY spectrum of Amycochromone F (**6**) (DMSO-d_6, 500 MHz)

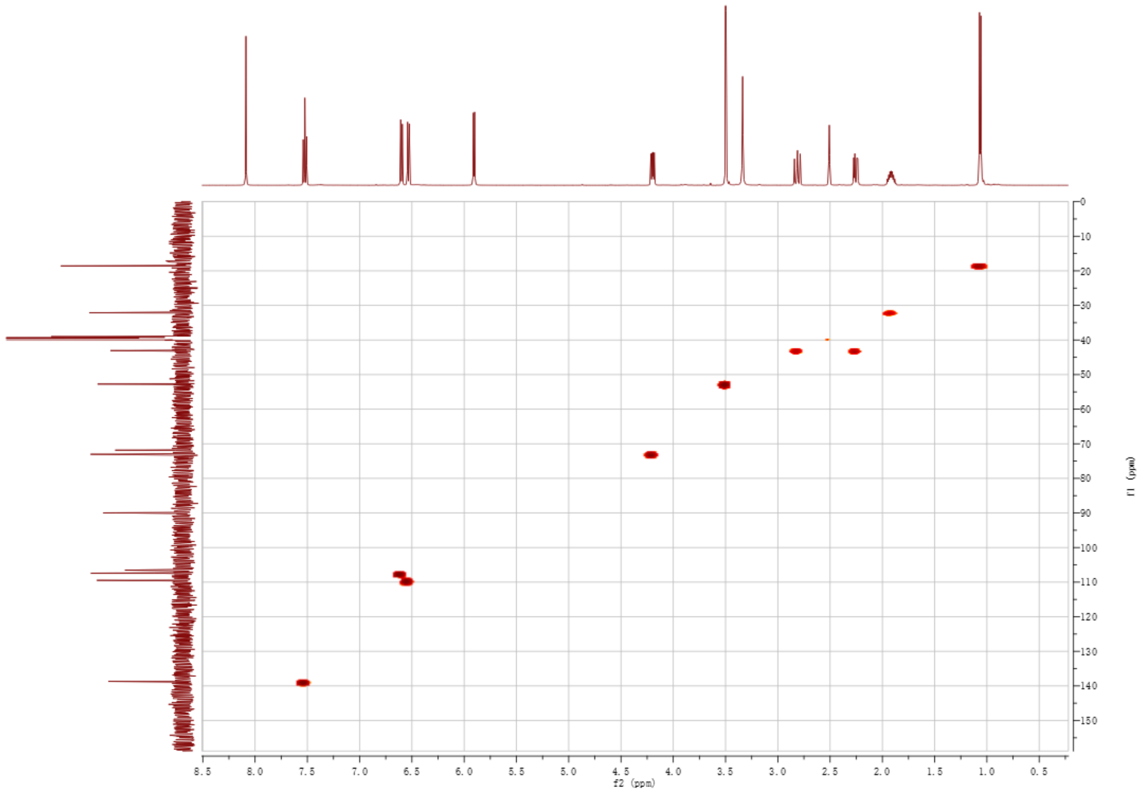

Figure S33. HSQC spectrum of Amycochromone F (**6**) (DMSO-d_6, 500 MHz)

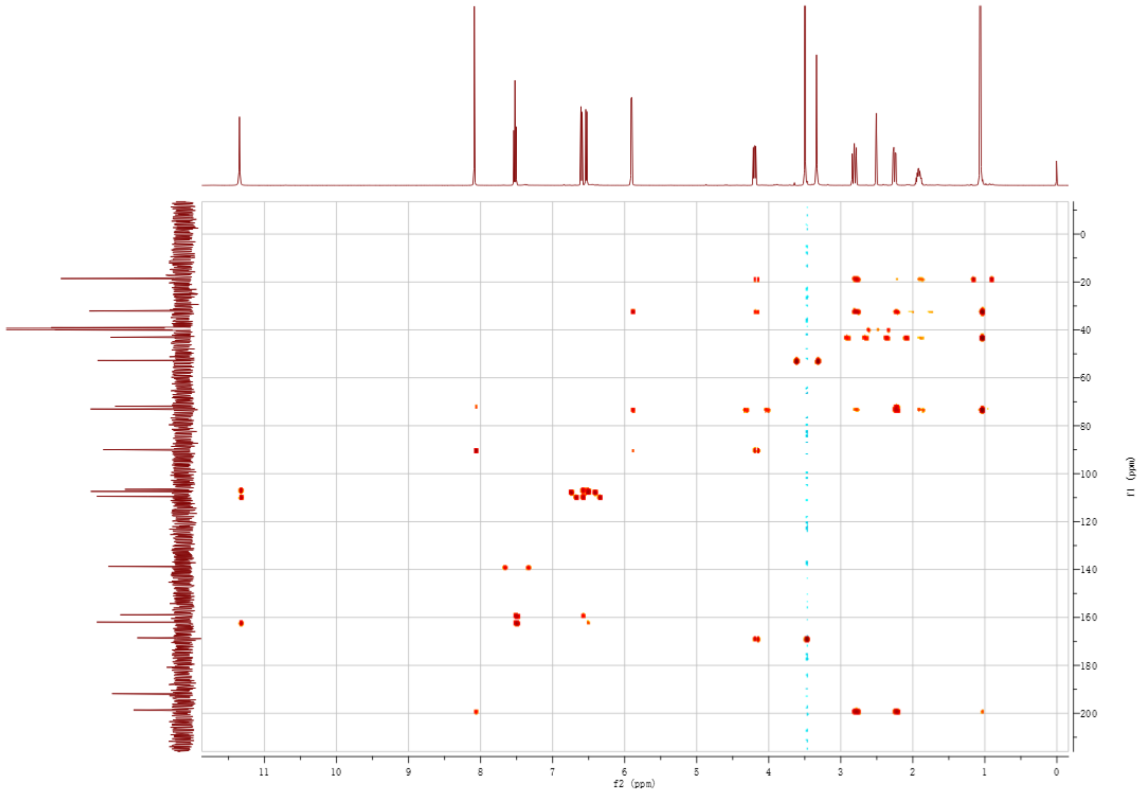

Figure S34. HMBC spectrum of Amycochromone F (**6**) (DMSO-*d*6, 500 MHz)

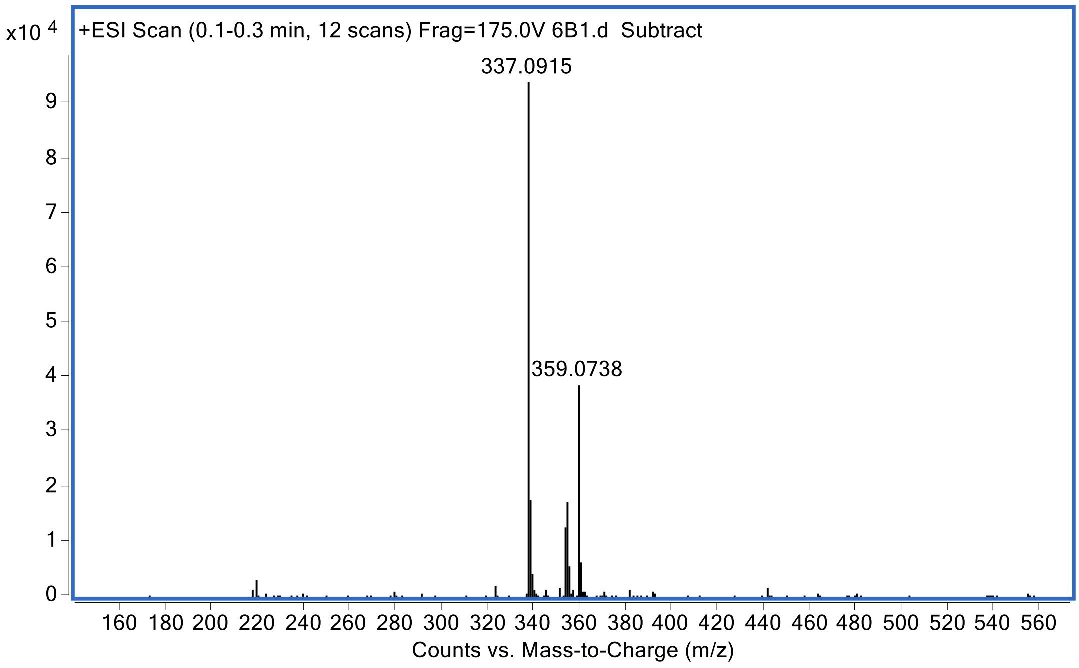

Figure S35. HRESIMS spectrum of Amycochromone F (**6**)

Table S1 Crystallographic data for compound **6**

Compound	**6**
Chemical formula	$C_{16}H_{16}O_8$, CH_4O, H_2O
Formula weight	386.34
Cystal size (mm)	0.006
Temperataur (K)	296.3(3)
radiation	0.71073
Cystal system	orthorhombic
Space group	P 21 21 21
aÅ	7.7760(3)
bÅ	8.6993(4)
cÅ	26.8196(11)
α°	90
β°	90
γ°	90
V (Å3)	1814.23(13)
Z	4
ϱCalcd (g/cm3)	1.414
F (000)	816
Absorp.coeff. (mm^{-1})	0.118
Range θ	3.038 to 26.368
Refins collected	18496(R= 0.0332)
independent reflections	3697
unique reflection with I > 2σ (I)	3133
Data/restr/paras	3697/ 0/ 255
GOF	1.011
R1/wR2[I > 2σ(I)]	0.0455/ 0.1177
R1/wR2[I>2σ(I)](all data))	0.0566 /0.1256
largest peak and hole (e Å-3)	0.560/ -0.231
